# Supplementary material for: All-natural 2D nanofluidics as highly-efficient osmotic energy generators
Source: Nat Commun. 2024 Apr 29;15:3649. doi: 10.1038/s41467-024-47915-z (PMC11058229; doi:10.1038/s41467-024-47915-z)
Supplement: Supplementary file 1 — Supplementary Information [file 41467_2024_47915_MOESM1_ESM.pdf]

Supplementary Information

All-natural 2D nanofluidics as highly-efficient osmotic energy generators

Jiadong Tang[1‡], Yun Wang[1‡], Hongyang Yang[2], Qianqian Zhang[1*], Ce Wang[1], Leyuan Li[1], Zilong Zheng[1*], Yuhong Jin[1], Hao Wang[1], Yifan Gu[2*], Tieyong Zuo[2]

1. Key Laboratory for New Functional Materials of Ministry of Education, Faculty of Materials and Manufacturing, Beijing University of Technology, Beijing 100124, P. R. China
2. Institute of Circular Economy, Faculty of Materials and Manufacturing, Beijing University of Technology, Beijing 100124, P. R. China
‡These authors contributed equally.

*Correspondence to: zhangqianqian@bjut.edu.cn; zilong.zheng@bjut.edu.cn; guyifan@bjut.edu.cn

The file includes:

Supplementary Notes 1-9

Supplementary Figures 1-38

Supplementary Tables 1-10

Supplementary References 1-73

Supplementary Notes

Supplementary Note 1: Calculation of the layer spacing

An example of studying the layer spacing of 2D-NNF, an intense peak can be detected at ~6.84° which is ascribed to the (002) peak corresponding to the interplanar spacing between the nanosheets[1]. According to Bragg's law:

$$2dsin\theta = n\lambda \tag{S1}$$

d is the layer spacing; θ is the angle between the incident wave and the scattering plane, λ is the wavelength (~0.154) of the incident wave and n=1. Here, 2θ (002) = 4.61° and the corresponding d (002) = 1.90 nm. So, the fully hydrated lamellar structure of the membrane is with a channel spacing of 0.90 nm considering of the thickness of single-layer MMT nanosheet. Therefore, according to the difference of the 2θ angle in ambient dried and fully hydrated of 2D-NNF, the layer spacing under different states can be obtained.

Supplementary Note 2: Calculation of the surface charge

The surface charge density (σ, C m^{-2}) of the 2D-NNF was calculated with the zeta potential according to the equation[2,3]:

$$\sigma = \frac{\varepsilon_0 \varepsilon_r \xi}{\lambda_D} \tag{S2}$$

where ε_0, ε_r, and ξ were the permittivity of a vacuum, the permittivity of water and zeta potential of 2D-NNF, respectively. λ_D is the Debye screening length, which can be obtained according equation[4]:

$$\lambda_D = \sqrt{\frac{\varepsilon_0 \varepsilon_r K_B T}{2 n_{bulk} z^2 e^2}} \tag{S3}$$

where k_B is the Boltzmann constant, and T is the absolute temperature; n_{bulk}, z and e represented the concentration of bulk solution, valence charge and elementary charge. For the solution containing same ions, the λ_D only depends the solution concentration. Thus, the varied solution concentration will cause the distortion of EDL, and a higher concentration leads to a thinner EDL (shorter λ_D) as demonstrated in Supplementary Table 1.

Supplementary Note 3: Cyclic voltammetry analyses

The cyclic voltammetry measurements were conducted with the electrochemical workstation (CHI-600E, Chenhua, Shanghai) with a three-electrode system. And the testing solution by mixing 5 mM $[Ru(NH_3)_6]^{3+}$ or $[Fe(CN)_6]^{3-}$ into 10 mM KCl solution (pH=7.0)[5]. The ITO-supported 2D-NNF as the working electrode, and the ITO-covered glass was washed with ethanol and H_2O before supporting. Besides, a platinum wire was used as the counter electrode and an Ag/AgCl wire was used as the reference electrode.

Supplementary Note 4: Electrode calibration

The energy conversion properties of the 2D-NNF are studied by measuring *I–V* curves in presence of a transmembrane salinity gradient. The Fig. 3b shows the equivalent circuit of the membrane. V_{OC}, V_{redox}, E_{diff}, and $R_{2D\text{-}NNF}$ represent the measured open-circuit voltage, the redox potential generated by the unequal potential drop at the electrode-solution interface, the diffusion potential contributed by the cation selective 2D-NNF, and the internal resistance of the membrane, respectively. The measured V_{OC} actually consists of two parts: V_{redox} and E_{diff}, which satisfy the following equation:

$$V_{OC} = V_{redox} + E_{diff} \qquad \text{(S4)}$$

In this work, the value of V_{redox} was measured using an experimental method. The 2D-NNF were replaced by a nonselective silicon membrane containing a single micro-window. In this case, the measured potential was contributed solely by the asymmetric redox reactions on the electrodes (V_{redox}). For the electrode calibration, the *I–V* curves were recorded by applying a sweeping voltage (−0.4 V, +0.4 V) at 20 mV steps.

Supplementary Note 5: Ion selectivity of 2D-NNF

The transference number t_n is calculated following the equation[6].

$$t_+ = \frac{1}{2}\left(\frac{E_{diff}}{\frac{RT}{zF}\ln\left(\frac{\gamma_{c_H} c_H}{\gamma_{c_L} c_L}\right)} + 1\right) \qquad \text{(S5)}$$

where t_+ is the cation transference number; E_{diff} refers to the diffusion potential; *R, T, z, F*, refer to the universal gas constant, absolute temperature, valence charge and Faraday constant, respectively; γ and *c* refer to ion activity coefficient and concentration.

The different solutions used in the work were KCl, NaCl, LiCl, $CaCl_2$ and $MgCl_2$ aqueous solution with different concentration. The ion activity coefficients of dilute solutions could be calculated using the simplified Debye-Hückel equation.

$$lg\gamma_\pm = -A \mid z_+ z_- \mid \sqrt{I} \qquad \text{(S6)}$$

where $\gamma_\pm$ is ion average activity coefficient, '*I*' is the ionic strength, *z* is ion valence number, and *A* is constant, the value is about 0.509 at 298 K. The calculated ion activity coefficients are summarized in the Supplementary Table 2.

Supplementary Note 6: Energy conversion efficiency

Normally, the energy conversion efficiency is defined as the ratio of the output energy (electrical energy) to the input energy (Gibbs free energy of mixing), the energy conversion efficiency corresponding to the maximum power generation (η_{max}) in the system can be calculated by the equation[7,8]:

$$\eta_{max} = \frac{1}{2}(2t_+ - 1)^2 \qquad \text{(S6)}$$

Supplementary Note 7: Molecular dynamics simulation

The molecular dynamics (MD) simulation in this study uses GROMACS (version 2020.3_GPU) software Berendsen thermostat, and the coupling time constant is 0.5 ps. The periodic boundary conditions and the ClayFF force field are used for calculation, the long-range electrostatic Particle-Mesh Ewald (PME) electrostatic calculation method is adopted. In this study, the short-range electrostatic and van der Waals cut off distance are set to 1 nm, and the concentration of K^+ and Cl^- ions is set to 1 mol L^{-1}.

Basis of modeling: The surface negative charge of MMT is mainly derived from the substitution of some trivalent Al^{3+} by divalent cations like Mg^{2+}, Fe^{2+} in the aluminum oxygen octahedron. The negative charge and layer spacing in 2D-NNF further increase compared to the pristine MMT. In order to simplify the model, the theoretical surface charge is close to the experimental value of 2D-NNF by adjusting the atomic ratio of Mg:Al, and the channel spacing was set according to the experimental value.

The transport of ions and charges is theoretically studied by MD simulation. The first is to establish a system with the theme of aqueous solution, at which time no ions exist. Step 2, replace some water molecules according to the concentration required by the experiment to make the ion concentration in the solution consistent with the experimental value, and set it as 1 mol L^{-1}. After NVT (N: number of particles, v: volume, t: temperature) balance at 450 K, NPT (N: number of particles, p: pressure, t: temperature) balance at 300 K for 5 ns annealing. The last step is to statistically analyze the ion migration number, ion migration speed and binding with VMD.

From the calculation results of MD, the water molecules near K^+ ion and Cl^- ion were obtained with DFT calculation at the level of M06-2X/def2-TZVPP, while the hydration were exported from MD equilibrium structure (Supplementary Fig. 19).

Supplementary Note 8: Details of technoeconomic analysis

we first analyzed the material flow in each stage from material procurement to laboratory exfoliation. The feeding quantity and yield were determined based on previous literature[9-13] and investigation of relevant production. To facilitate calculation, material losses during intermediate processes were not take into account, ensuring the maintenance of material balance before and after. The cost of raw materials and chemical reagents was estimated using the ton price of industrial grade purity reagents (Supplementary Table 9), supplemented by the main reference survey website: (https://www.100ppi.com/, https://www.mysteel.com/, https://p4psearch.1688.com/).

The prices of raw materials and chemical reagents in different periods will fluctuate. For labor costs, equipment depreciation, and other fixed costs were not considered at present. When calculating the costs of materials, reagents and energy consumption, the price differences MMT, GO, and MXene powder were reasonable and close to the enterprise prices provided through our investigated. Of course, this value was mainly

for reference and guidance, and the actual costs must be determined after a large-scale practical production. Additionally, energy consumption was worth concerning. To make the estimation of energy consumption close to reality, we made estimates based on the equipment specifications and required service time for small-scale production in experimental and industrial settings.

To conduct an environmental impact assessment, the life cycle assessment (LCA) method was used to quantify the environmental impact of five production lines: MMT, GO, MXene, ANF and CNF (Supplementary Table 8). This assessment involved determining the input and output amounts of materials (Supplementary Table 7) using GaBi software (an environmental impact analysis software designed in accordance with the principles of LCA methodology was jointly developed by the LBP Institute of the University of Stuttgart and PE Company), including index: GWP (global warming potential), AP (acidification potential), ADP (abiotic depletion fossil), HTP (human toxicity potential), MAETP (marine aquatic eco-toxicity potential), FAETP (freshwater eco-toxicity potential), POCP (photochemical ozone creation potential), EP (eutrophication potential).

For Supplementary Table 10, detailed production processes of 2D materials and nanocellulose used for nanofluidics preparations in reported works[1,14-17] were summarized to evaluate the resource, environment and economic impacts of the mainstream 2D material-based nanofluidics.

Supplementary Note 9: TOPSIS calculation method

According to the different decision-making objectives, the environment, resource, and economy factors are coupled through the method of multi-objective decision-making to ensure the objectivity of target weight setting. Among the multi-objective decision-making methods, the TOPSIS method is to establish the initial decision matrix and standardize it. By calculating the distance between each scheme relative to the optimal and worst scheme, the closeness between each scheme and the optimal scheme is obtained. Its dimensionless method for indicators can effectively solve the incommensurability between environment, resource, and economy factors, and the construction of positive and negative ideal schemes can effectively achieve the purpose of scheme sorting and selection.

(1) The multi-objective decision analysis model is constructed based on TOPSIS method

The basic principle of TOPSIS is to sort the existing schemes according to the proximity between the evaluation object and the idealized target to determine the optimal scheme. Among them, the positive and negative ideal solutions are assumed as the best and worst schemes. The positive ideal solution refers to an ideal solution that each index reaches the optimal; while the negative ideal solution is the worst case for each index

value. The evaluation steps of TOPSIS multi-objective decision-making method are as follows:

Suppose that there are several schemes: $R = \{\mathbf{r_1}, \mathbf{r_2}, ..., \mathbf{r_m}\}$, each scheme has $f = \{\mathbf{f_1}, \mathbf{f_2}, ..., \mathbf{f_n}\}$ evaluation indexes, and the initial matrix is as follows:

$$R = (f_{ij})_{m\times n} = \begin{vmatrix} f_{11} & f_{12} & \cdots & f_{1n} \\ f_{21} & f_{22} & \cdots & f_{2n} \\ \cdots & \cdots & \cdots & \cdots \\ f_{m1} & f_{m2} & \cdots & f_{mn} \end{vmatrix}$$

where m and n are the number of solutions, the dimension of the evaluation index. Here n=3, refers to the three evaluation indexes of environment, resource, and economy, respectively.

① Standardized decision matrix: to eliminate the incommensurability between indicators.

$$r_{ij} = \frac{f_{ij}}{\sqrt{\sum_{i=1}^{m} f_{ij}^2}} \quad (i = 1,2,\dots,m;\ j = 1,2,\dots,n)$$

where r_{ij} is standardized decision matrix. The environment, resource, and economy factors can be compared objectively after the standardization process.

② Weighted standardized decision matrix: the decision indexes are weighted respectively to obtain the weighted standardized decision matrix.

$$V_{ij} = r_{ij} \times \mathbf{w_j} \quad (i = 1,2,\dots,m;\ j = 1,2,\dots,n)$$

where V_{ij} refers to the weighted normalized decision matrix, w_j refers to the weight of each decision index f_j, $\sum_{j=1}^{n} \mathbf{w_j} = 1$.

③ Calculate positive and negative ideal solutions.

$$A^+ = \{\mathbf{v_1^+}, \mathbf{v_2^+}, \dots, \mathbf{v_n^+}\} = \{(max\mathbf{v_{ij}}|j \in I'), (min\mathbf{v_{ij}}|j \in I'')\}$$

$$A^- = \{\mathbf{v_1^-}, \mathbf{v_2^-}, \dots, \mathbf{v_n^-}\} = \{(max\mathbf{v_{ij}}|j \in I'), (min\mathbf{v_{ij}}|j \in I'')\}$$

where A^+ and A^- are the positive and negative ideal solutions. For the revenue index, the maximum value of the row vector is a positive ideal solution, while the opposite is true for cost-based indexes. In this study, because the target scheme is to achieve the lowest resource consumption, environmental impact and the economic cost, the three evaluation indexes are all cost-based, that is, the minimum value of the row vector is a positive ideal solution.

④ Calculate the distance between the superior and inferior solutions, and sort the schemes:

$$d_i^+ = \sqrt{\sum_{j=1}^{n} (\mathbf{v_{ij}} - \mathbf{v_j^+})^2} \ \ (i = 1,2,\dots,m)$$

$$d_i^- = \sqrt{\sum_{j=1}^{n} (\mathbf{v_{ij}} - \mathbf{v_j^-})^2} \ \ (i = 1,2,\dots,m)$$

where d_i^+ refers to the distance between the evaluation scheme and the positive ideal solution, d_i^- refers to the distance between the evaluation scheme and the negative ideal solution.

According to the r_i* score from high to low, the final ranking of the schemes can be considered as the best scheme.

$$r_i = \frac{d_i^-}{d_i^+ + d_i^-} (i = 1,2,\dots,m)$$

(2) Model sensitivity analysis

Based on the above TOPSIS method, the sensitivity analysis is further integrated to calculate the change level of the final results of environment, resource, and economy factors under different weight distribution modes, and to investigate the stability of the evaluation results. When the weights of environment, resource, and economy factors change from 0 to 1, the ranking of different schemes is recalculated to further determine the stability interval of the evaluation results (Fig. 6d).

Supplementary Figures

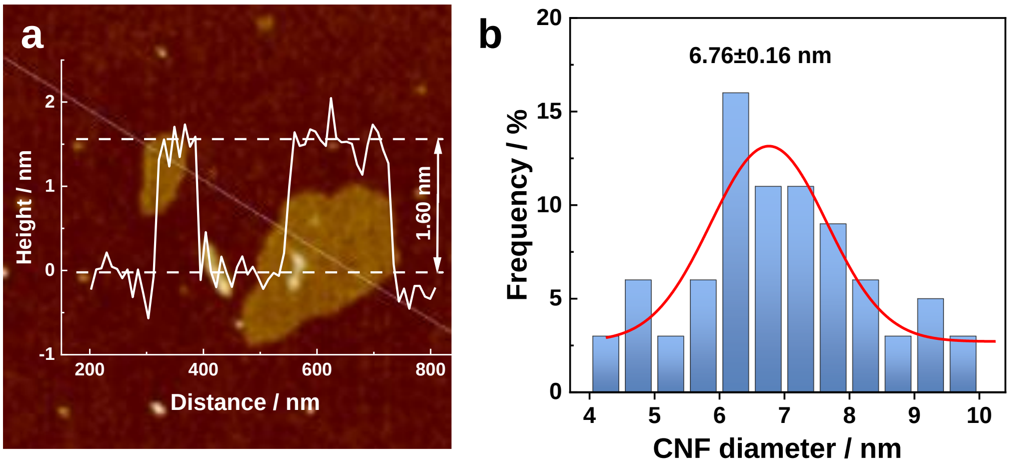

Supplementary Fig. 1 (a) AFM images and corresponding height profiles of MMT nanosheets. (b) Diameter distributions of cellulose nanofibers. Source data are provided as a Source Data file.

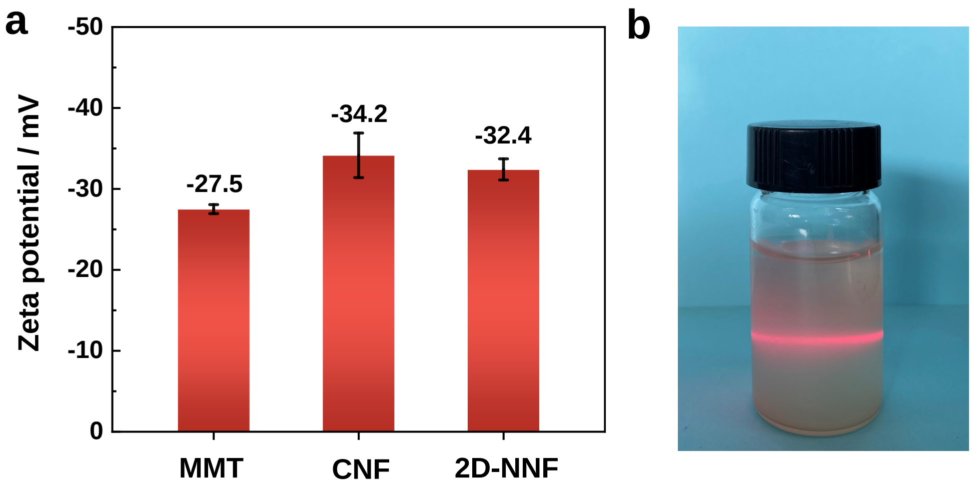

Supplementary Fig. 2 (a) The surface zeta potential comparison of MMT, CNF and 2D-NNF. (b) The Tyndall scattering effect of the as-prepared 2D-NNF colloidal suspension, indicating the good dispersion in water. The error bars in the figure represent the standard deviations for six measurements.

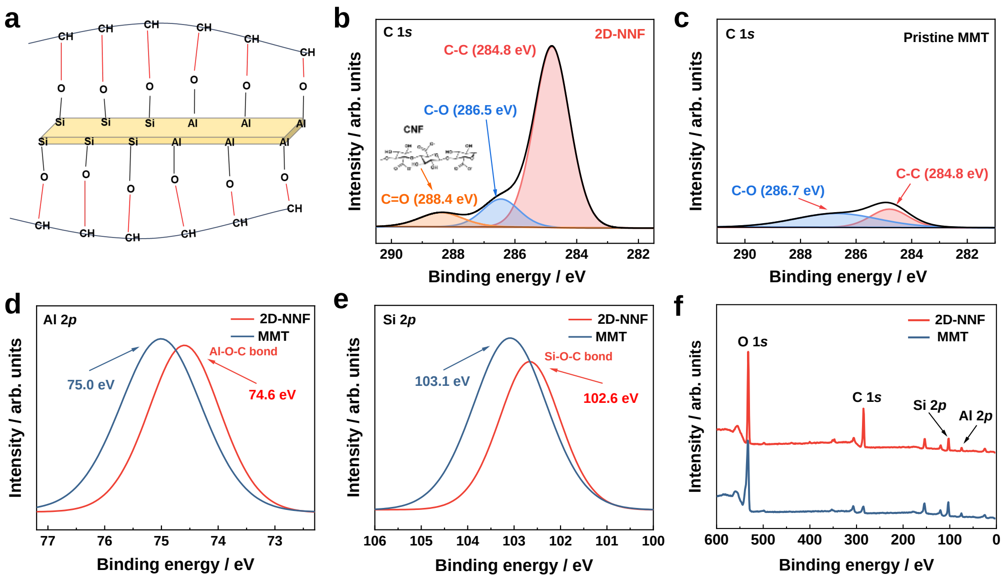

Supplementary Fig. 3 (a) Schematic of the covalent bonds formed between Si-OH or Al-OH at the edge of MMT and CNFs. XPS spectra of C 1*s* of (b) 2D-NNF and (c) pristine MMT nanofluidics. The appearance of C=O peak (288.4 eV) of 2D-NNF compared with pristine MMT confirmed the existence of CNFs. Also, there is a remarkable increase in the peak areas of C-O and C-C with the introduction of CNFs. High spectra of 2D-NNF and MMT for (d) Al 2*p* and (e) Si 2*p* region. The characteristic peak Si 2*p* originated from Si-O-Si/Si-O-Al, and the main peak of Al 2*p* was attributed to the Al-OH and Al-O-Si. The binding energy displacement of Al 2*p* and Si 2*p* compared with pristine MMT membrane, which was mainly attributed to the formation of covalent bonds between MMT and CNFs. (f) The survey XPS of 2D-NNF and MMT nanofluidics. For the 2D-NNF, C 1*s* and O 1*s* peak enhancement due to the introduction of CNFs. For pristine MMT nanofluidics, the appearance of carbon may be due to surface adsorption with weak strength and there is no obvious C=O peak in C 1*s* region. Source data are provided as a Source Data file.

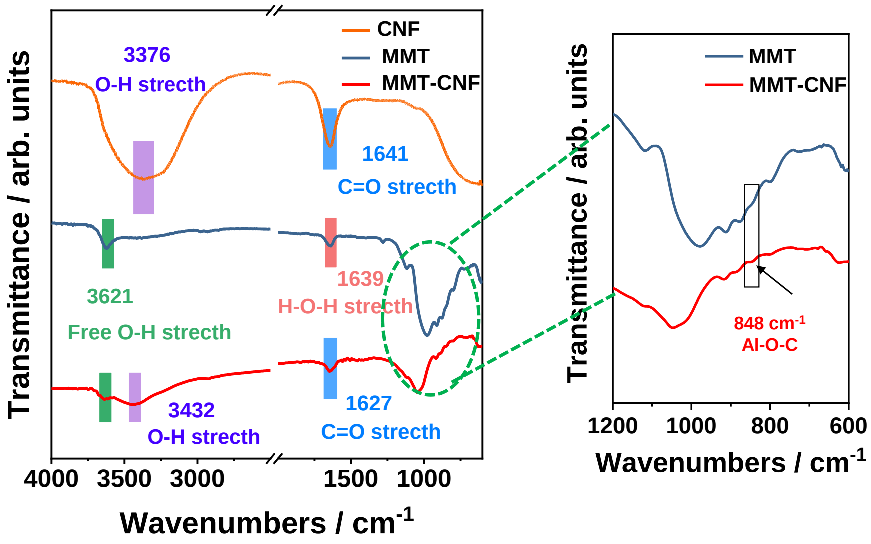

Supplementary Fig. 4 FTIR spectrum of CNF, pristine MMT nanofluidics and 2D-NNF, the marked characteristic peaks correspond to the functional groups. The enhanced emerged peak at 3432 cm^{-1} and 1627 cm^{-1} corresponds to -OH and C=O group vibrations, respective, which confirmed the successful introduction of CNFs. Besides, the decrease of free OH on the surface of 2D-NNF at 3621 cm^{-1} indicates the occurrence of dehydration condensation reaction. Source data are provided as a Source Data file.

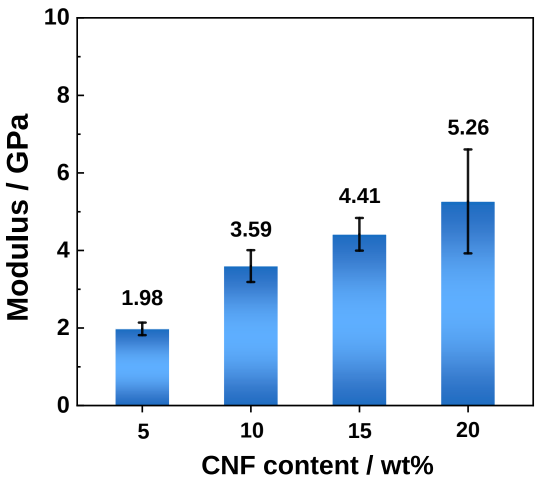

Supplementary Fig. 5 The Young's modulus of the 2D-NNF with different CNF contents. The error bars in the figure represent the standard deviations for five measurements.

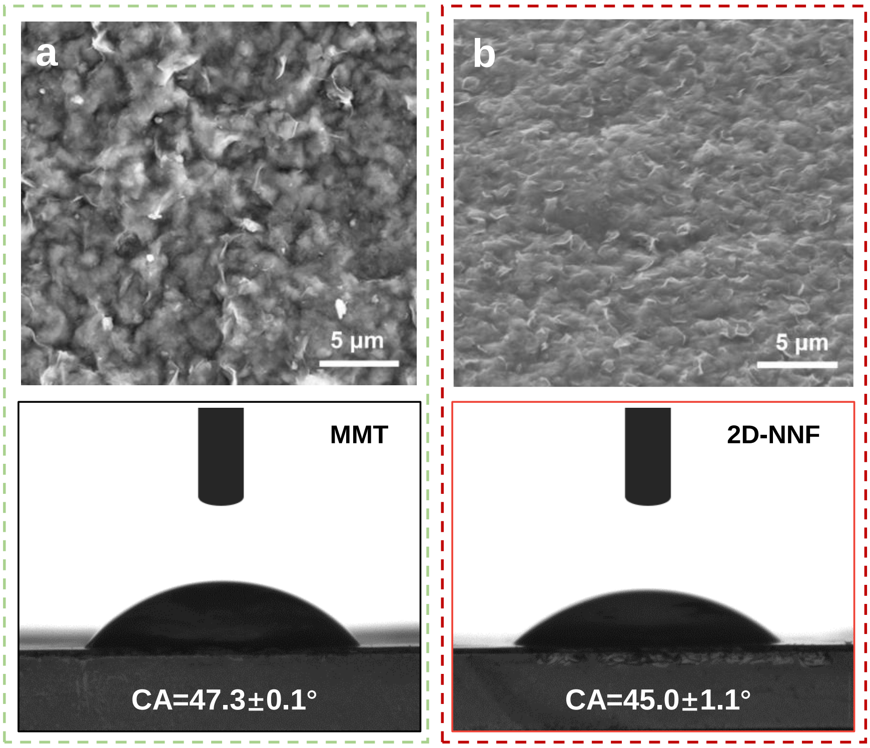

Supplementary Fig. 6 Top-view SEM images and surface contact angles of KCl electrolyte for (a) MMT nanofluidics and (b) 2D-NNF.

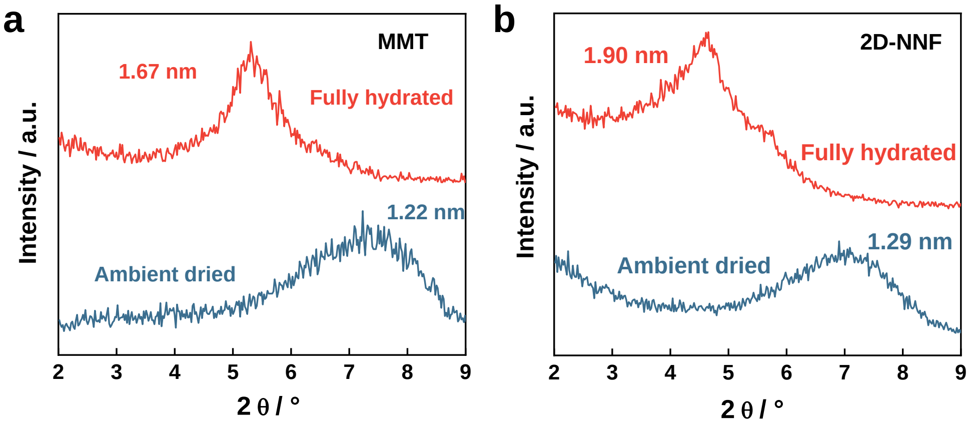

Supplementary Fig. 7 Variation of nanochannel space of (a) pristine MMT nanofluidics and (b) 2D-NNF (10%-CNF content) under ambient dried and fully hydrated conditions at room temperature, indicating that the introduction of nanofibers enlarges the nanochannel space. Source data are provided as a Source Data file.

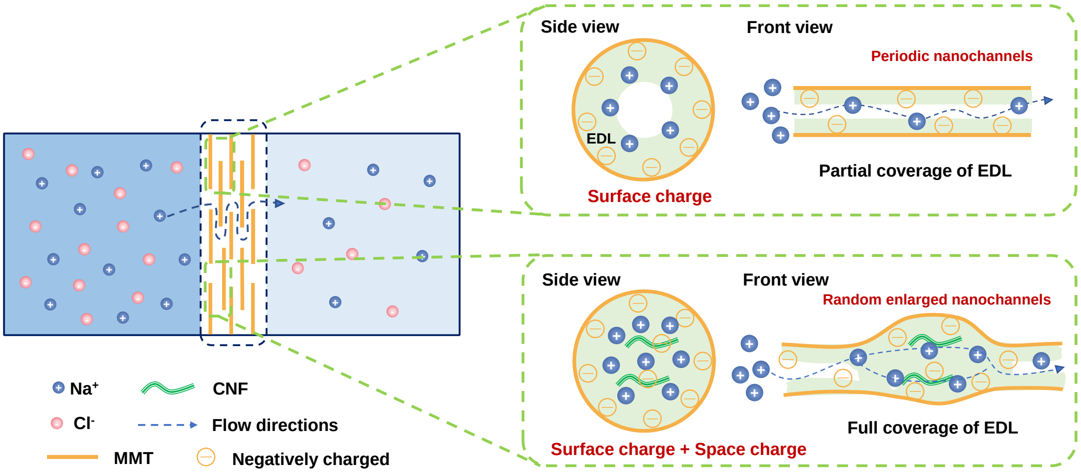

Supplementary Fig. 8 Schematic diagram for the microstructure of the 2D-NNF with periodic interlayer nanochannels and random enlarged nanochannels with CNFs intercalation, and surface charge and space charge were incorporated into the interlamellar nanochannels. The CNFs carry abundant surface negative charges, which could enhance the local space charge density in the enlarged nanochannels[31]. Both the surface charge and space charge could form EDLs for selective transport of cations. For the pristine MMT nanochannels with only surface charge, the formed EDL region may not fully cover the whole channels, especially for the high-concentration solutions, which reduces the cation selectivity and thus affects the osmotic energy harvesting. When introducing the space charge of CNFs, the EDL region was enlarged and covered almost the whole nanochannels, contributing to the enhanced ion selectivity and thus an improved osmotic energy harvesting.

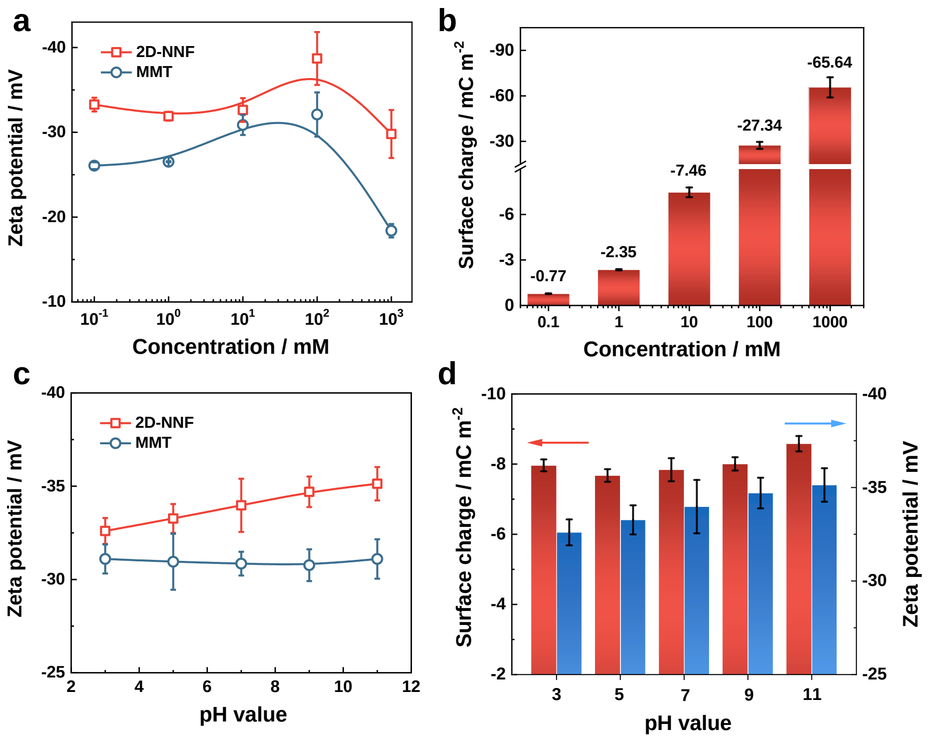

Supplementary Fig. 9 (a) The surface zeta potential measurement of MMT and 2D-NNF at various KCl electrolyte concentration, suggesting that the negative charge of 2D-NNF is higher than MMT in the whole concentration range from 0.1 mM to 1000 mM. (b) The surface charge as a function of concentration for KCl at ambient temperature. (c) Zeta potential of pristine MMT and 2D-NNF and (d) the surface charge of the 2D-NNF as a function of pH value of 10 mM KCl solution at ambient temperature. The surface charge of MMT crystal includes the excess permanent charge (90%) and few variable charge (10%)[18,19]. The permanent charge is generated by isomorphic substitution of MMT crystal. The structural unit of MMT is a layer of aluminum oxide octahedron sandwiched between two layers of silica tetrahedron. The substitution of high valence ions (Si^{4+}, Al^{3+}) by low valence ions (Mg^{2+}) results in the formation of surface negative charge. The polarity and density of such charge could not be affected by the solution conditions[20]. In the MMT, the permanent negative charges are 90 percent of the total charge, which determines the charge polarity and density of the materials. As a result, the zeta potential of MMT remained stable with varied solution pH. For the CNF, the surface charge is generated from the protonation or deprotonation of excess carboxyl groups, which thus carries negative charges in the pH range of 3~11. Therefore, the introduction of CNF obviously enhances the negativity of zeta potential of MMT, and the value varied with solution pH. All the error bars in the figure represent the standard deviations for six measurements. Source data are provided as a Source Data file.

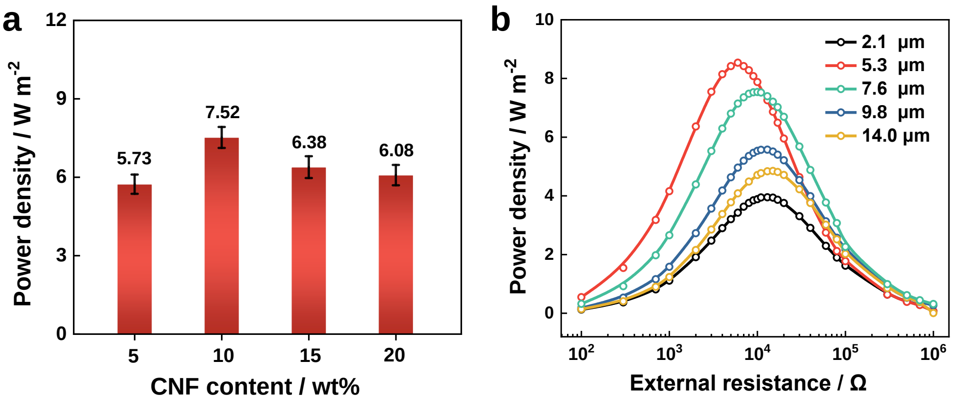

Supplementary Fig. 10 Osmotic energy conversion performance under an artificial seawater and river water salinity gradient. (a) Influence of the CNFs weight content and (b) the membrane thickness on osmotic energy generation. The above results confirm that reduce the membrane thickness can improve the power density due to the decreased internal resistance and the shorten ion diffusion path of the 2D-NNF, but when the membrane thickness was further reduced to 2.1 μm, the membrane became fragile and less dense, results in a power density as low as 3.95 W m^{-2} owing to the lower ion permeability. The error bars in the figure represent the standard deviations for five measurements. Source data are provided as a Source Data file.

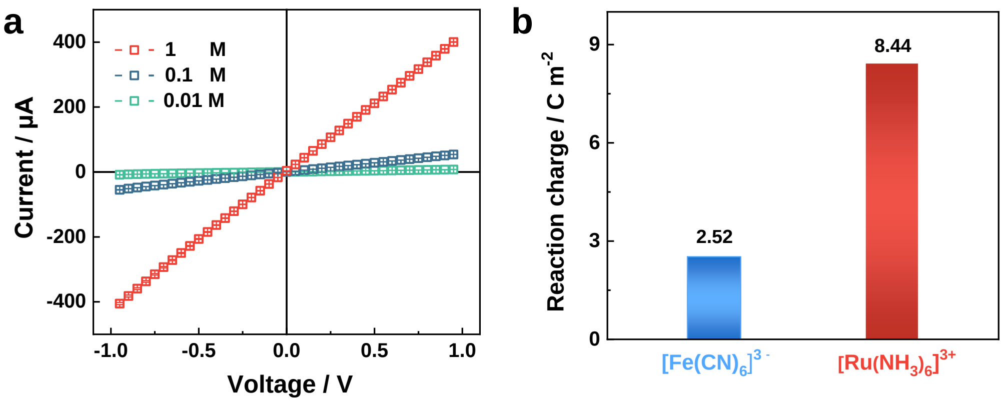

Supplementary Fig. 11 (a) *I–V* curves of 2D-NNF recorded in KCl electrolyte (PH~5.7) with the concentration from 0.01 to 1 M. The error bars in the figure represent the standard deviations for three measurements. (b) Reaction charges corresponding redox peak area when using $[Ru(NH_3)_6]^{3+}$ and $[Fe(CN)_6]^{3-}$ probes. Source data are provided as a Source Data file.

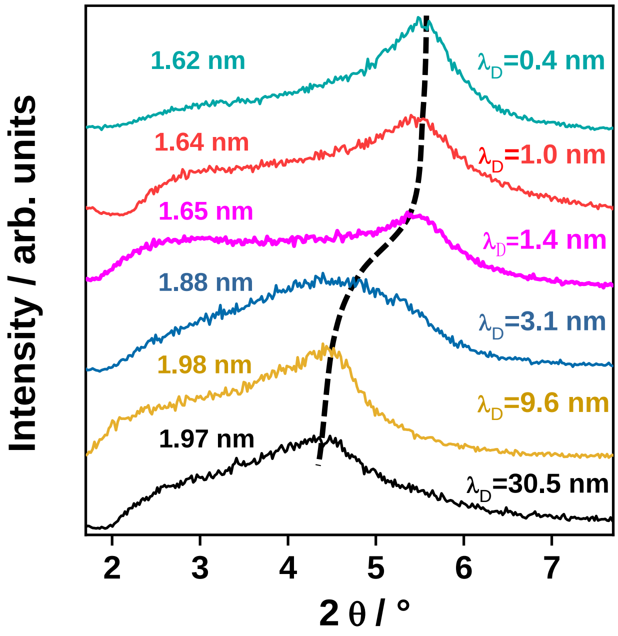

Supplementary Fig. 12 Evolution of layer spacing (left) of the hydrated 2D-NNF in the applied solutions with different Debye lengths (right). The layer spacing was measured with small-angle XRD. Before the measurement, the 2D-NNF membrane was immersed in KCl solution with different concentration for 24 h and then wiped the residual solution on the surface of membrane. The layer spacing increases slightly with the increasing λ_D due to the enhanced thickness of EDL (equal to λ_D). Source data are provided as a Source Data file.

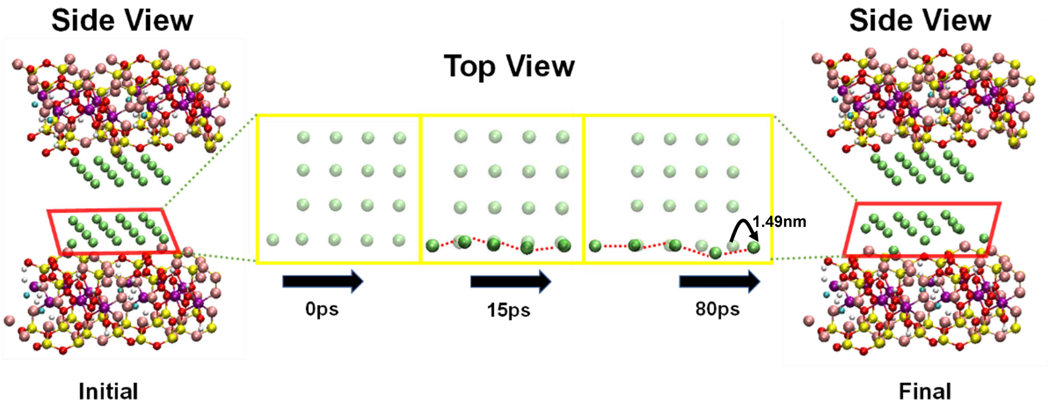

Supplementary Fig. 13 Ab initio molecular dynamics simulations of rapid K^+ transport in diffuse layer according to hopping mechanism.

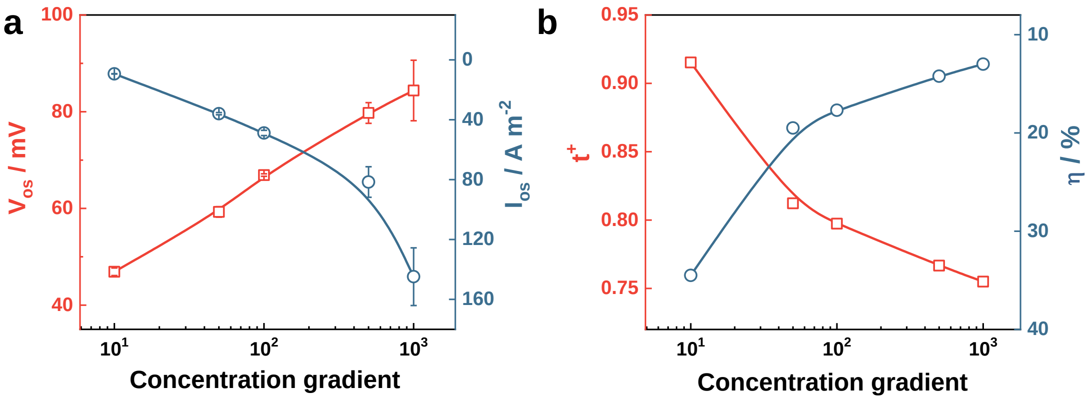

Supplementary Fig. 14 (a) The recorded diffusion potential and diffusion current under a series of KCl concentration gradients, where the low concentration side is fixed at 1 mM. The error bars in the figure represent the standard deviations for three measurements. (b) The calculated transmembrane ion transference number (t^{+}) and energy conversion efficiency (η) under the same range of electrolyte concentrations. Source data are provided as a Source Data file.

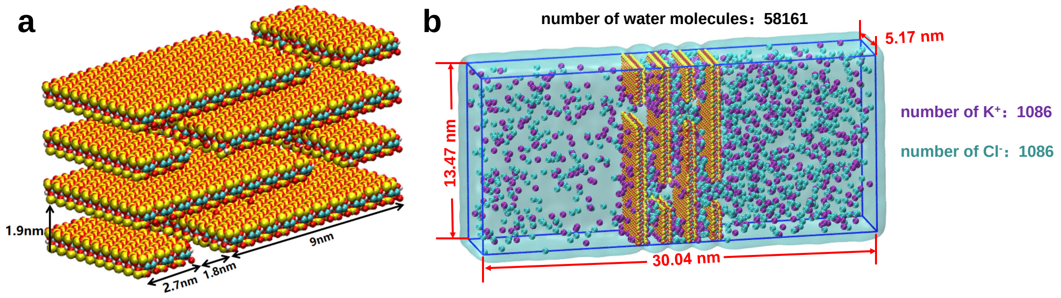

Supplementary Fig. 15 (a) The simulation model of a single slit of 2D-NNF flakes. The flake space is set according to the experimental value (Supplementary Fig. 7). (b) The parameters in the MD simulations.

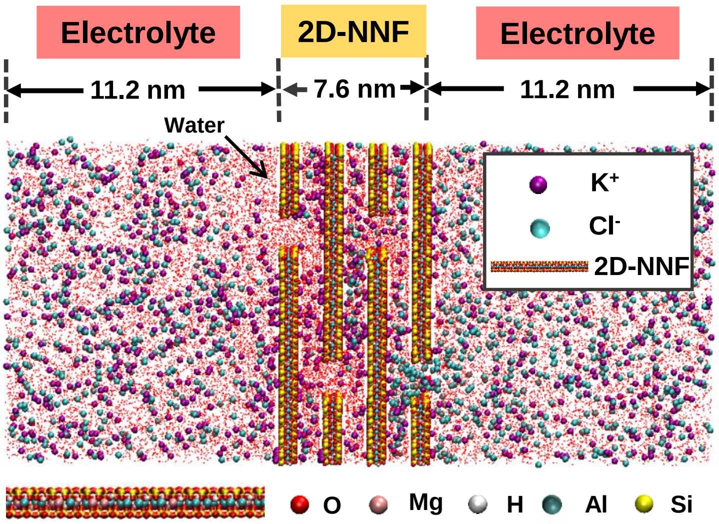

Supplementary Fig. 16 The theoretical model for MD simulations consists of 2D-NNF between two cell electrolyte layers. A 7.6 nm-thick nanosheet layer within the black dotted lines represents the major part of the membrane for the calculations. The model part demonstrates the distribution of ions at the 15th ns after diffusion equilibrium.

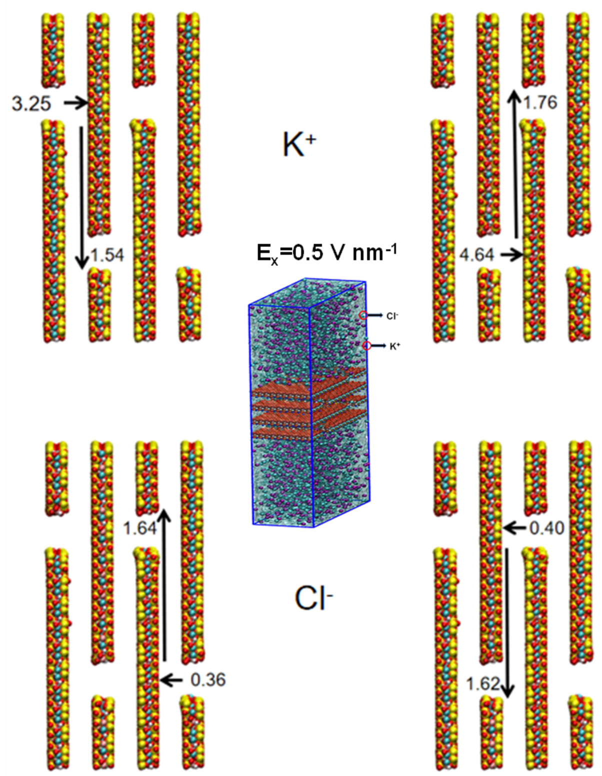

Supplementary Fig. 17 Evolution of the vertical and horizontal transport rates (m s^{-1}) of K^+ ions and Cl^- ions were calculated under a transmembrane electric field of 0.5 V nm^{-1} (inset).

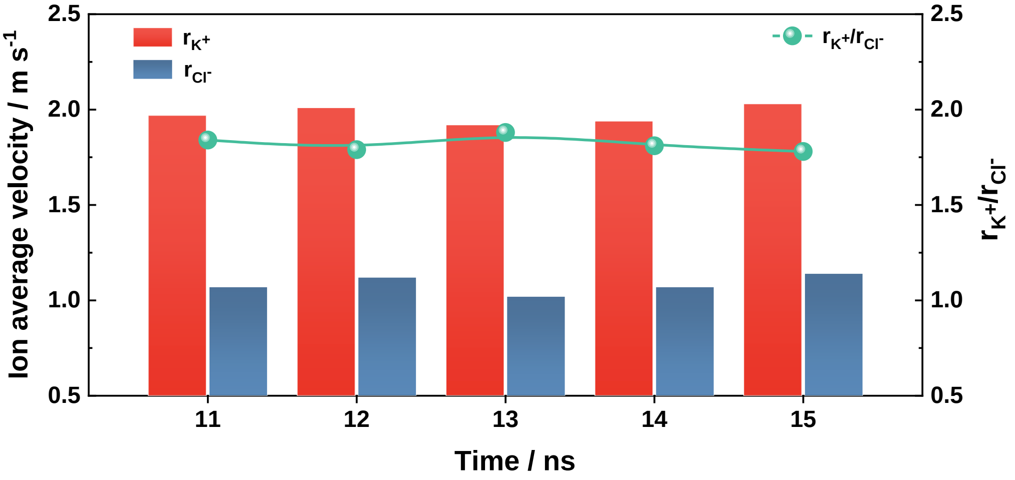

Supplementary Fig. 18 Evolution of the calculated ratio between average transmission rate of K^+ ions and Cl^- ions over time after diffusion equilibrium. Source data are provided as a Source Data file.

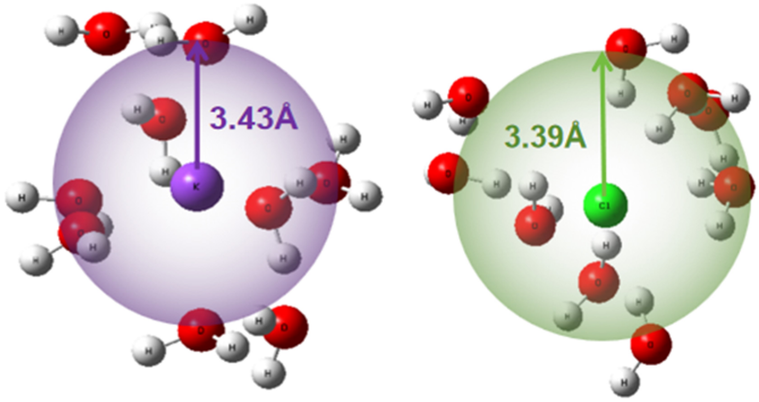

Supplementary Fig. 19 Configurations for the hydration of K^+ ion (purple) and Cl^- ion (green) exported from MD calculations. In aqueous environment, the cluster of hydrated K^+ with water molecules indeed has a much larger radius (3.43 Å) than that of proton, considering additional K-O coordinate bond. The K ions undergo a dehydration process before entering the nanochannel, while the ionic radius of K^+ following dehydration is 1.33 Å. Moreover, DFT calculations indicated the hydration energy of K^+ and Cl^- are 3.07 eV and 3.72 eV, respectively, while the Cl^- hydration is based H-Cl hydrogen bond. Therefore, K^+ is easier to enter the nanochannel compared with Cl^-, given low hydration energy.

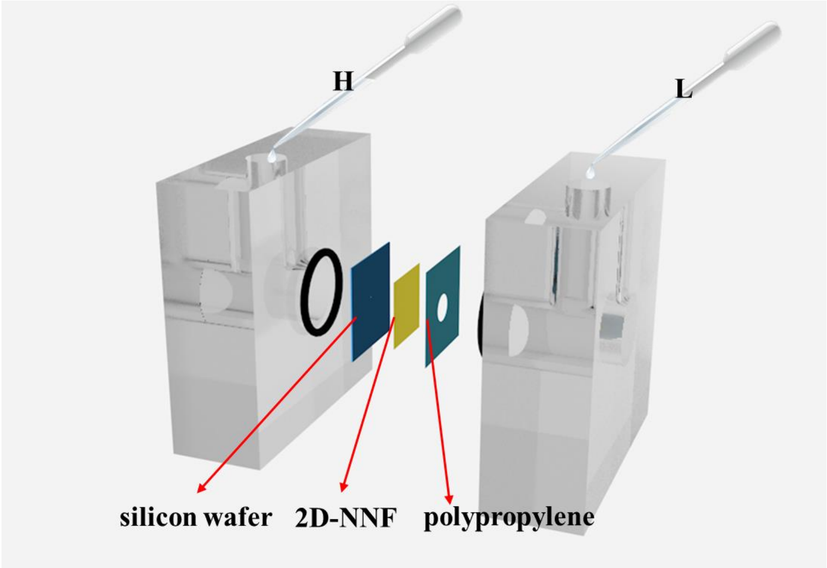

Supplementary Fig. 20 Schematic diagram of the concentration cell system, which contains a perforated silicon wafer, 2D-NNF, and a perforated polypropylene protective layer.

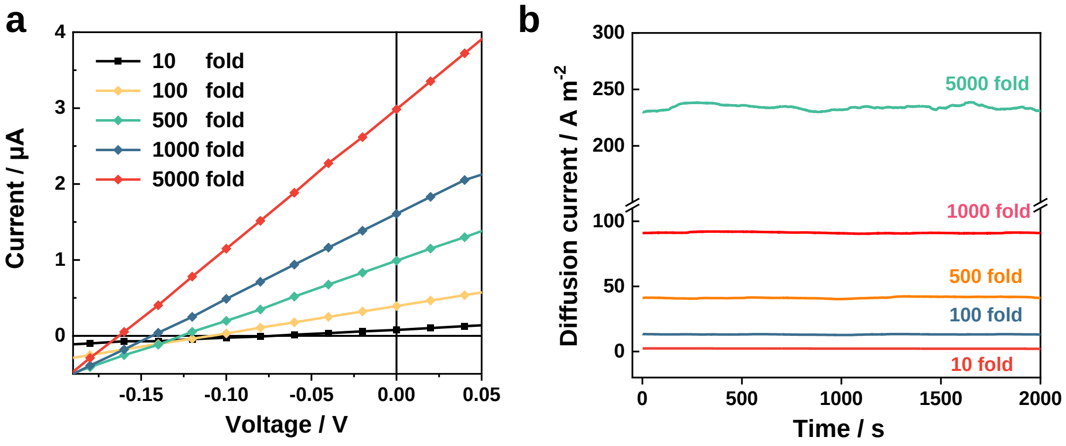

Supplementary Fig. 21 (a) *I–V* curves under varied NaCl concentrations gradients, with the high-concentration (HC) side was ranging from 1 mM to 0.5 M, while the low-concentration (LC) side was fixed at 0.1 mM. (b) Diffusion current density-time curves under the same range of electrolyte concentrations. Source data are provided as a Source Data file.

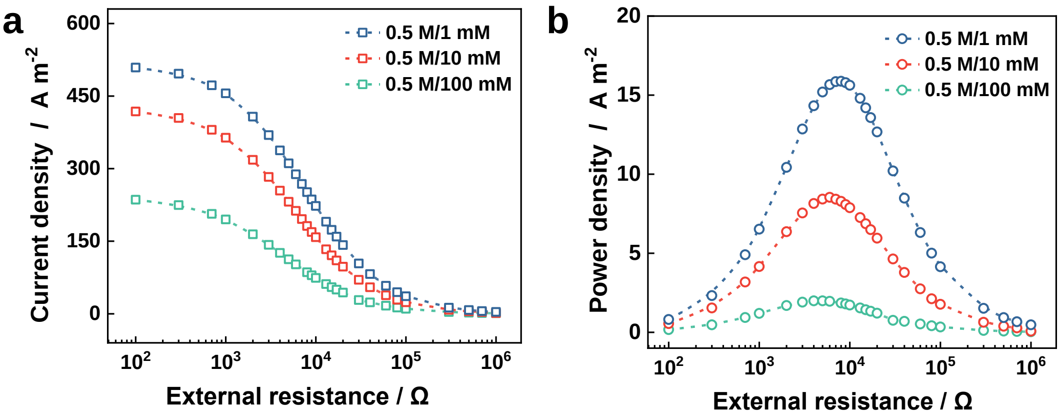

Supplementary Fig. 22 (a) Under three salinity gradients, the measured current densities all gradually decrease with the increasing external resistance. (b) The corresponding output power achieves the maximum values of 1.99, 8.61, and 15.84 W m^{-2}, respectively, for the 5-fold, 50-fold, and 500-fold salinity gradient (NaCl). Source data are provided as a Source Data file.

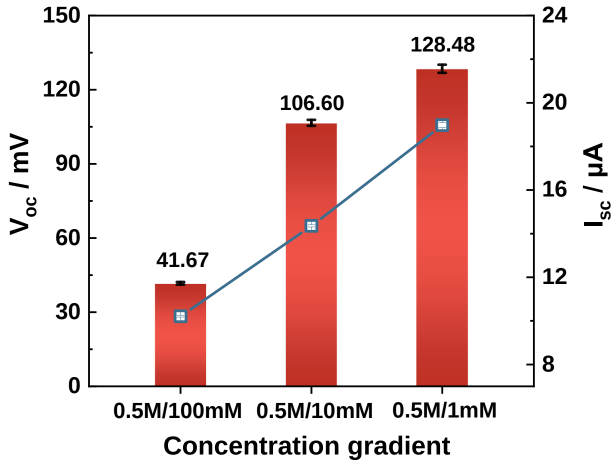

Supplementary Fig. 23 Open-circuit voltage (V_{OC}) and short-circuit current (I_{SC}) of 2D-NNF under different concentration gradient (NaCl). The error bars in the figure represent the standard deviations for six measurements.

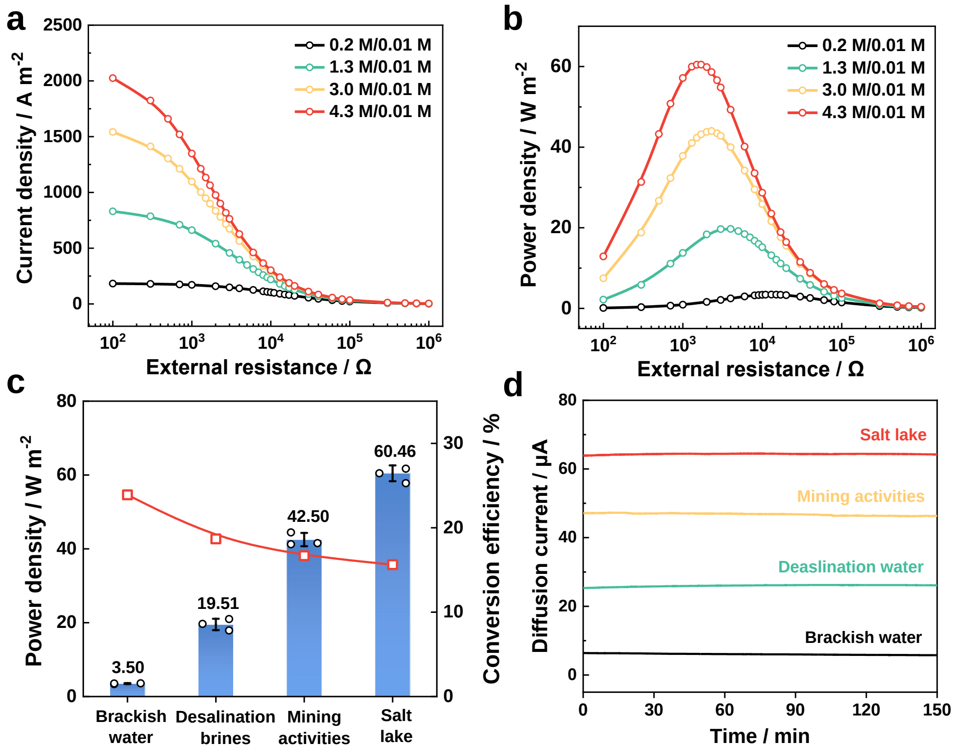

Supplementary Fig. 24 Various salinities that simulate different water environments were constructed to study the performance of 2D-NNF under high polarization conditions. The artificial seawater was replaced by artificial brackish water (0.2 M), desalination brine (1.3 M), mining waste water (3.0 M) and salt-lake water (4.3 M). (a) The current densities and (b) power densities at different salinity conditions as a function of the external resistance. Power generation and conversion efficiency (c) and long-term current output (d) under a series of artificial water resources including brackish water, desalination brines, brines from mining activities, and water from salt-lake. The output current and power density of 2D-NNF gradually increase with the salinity gradient, while the energy conversion efficiency decreases slightly given the ionic concentration polarization. The error bars in the figure represent the standard deviations for three measurements. Source data are provided as a Source Data file.

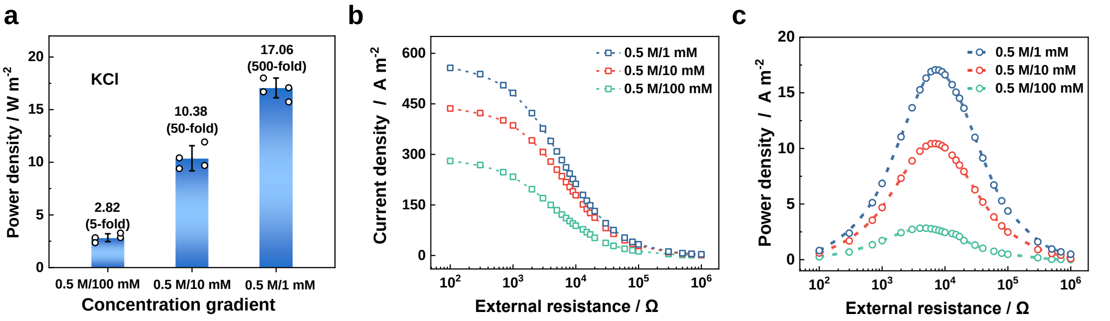

Supplementary Fig. 25 (a) The output power densities at different concentration folds. The high-salinity solution is fixed at 0.5 M KCl, and low-salinity is varied from 1 mM to 100 mM. The maximum value is 17.06 W m^{-2}. (b) The current densities and (c) power density at different concentration folds as a function of the external resistance. The error bars in the figure represent the standard deviations for four measurements. Source data are provided as a Source Data file.

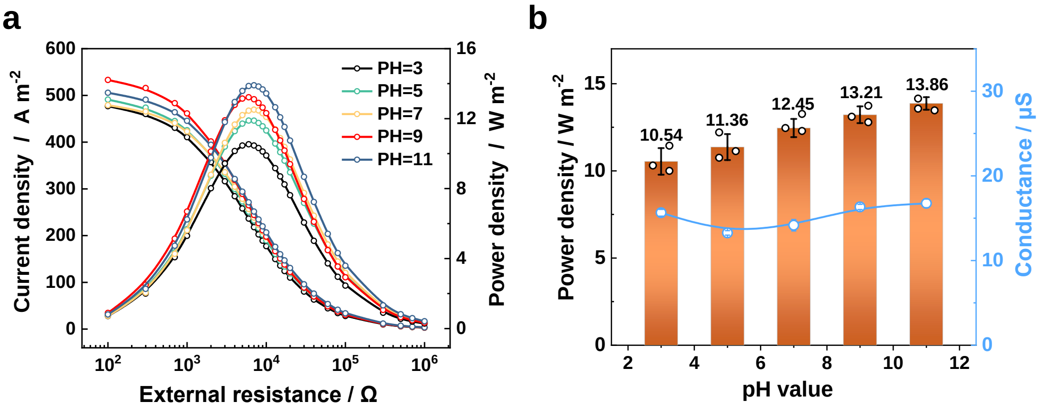

Supplementary Fig. 26 (a) The current density and power density of 2D-NNF at different pH values. The salinity electrolyte system is 0.5 M/0.01 M KCl. (b) Maximum output power density of 2D-NNF under different pH values, and conductance of 2D-NNF in 0.01 M KCl with different pH values. The error bars in the figure represent the standard deviations for three measurements. Source data are provided as a Source Data file.

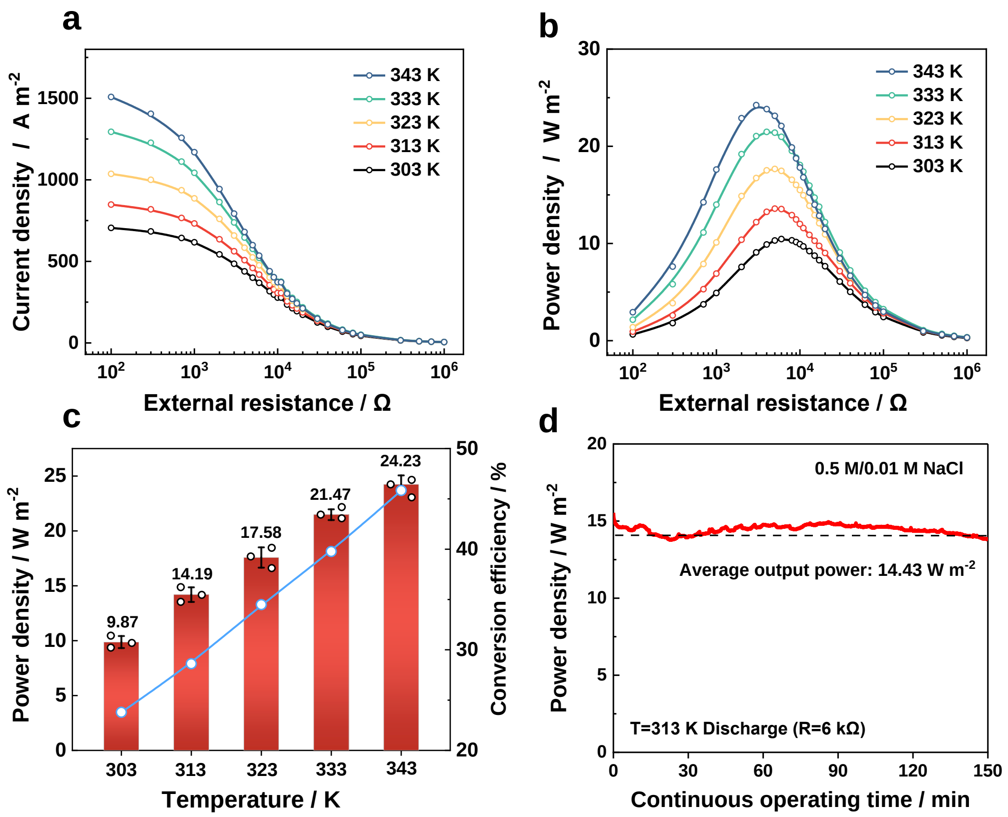

Supplementary Fig. 27 (a) Current density and (b) power density output at different temperature. (c) Maximum power generation and energy conversion efficiency under different temperature (concentration gradient is 0.5 M/0.01 M NaCl). (d) Lifetime of power output stability under the load resistance of ~6 kΩ at 313 K. The error bars in the figure represent the standard deviations for three measurements. Source data are provided as a Source Data file.

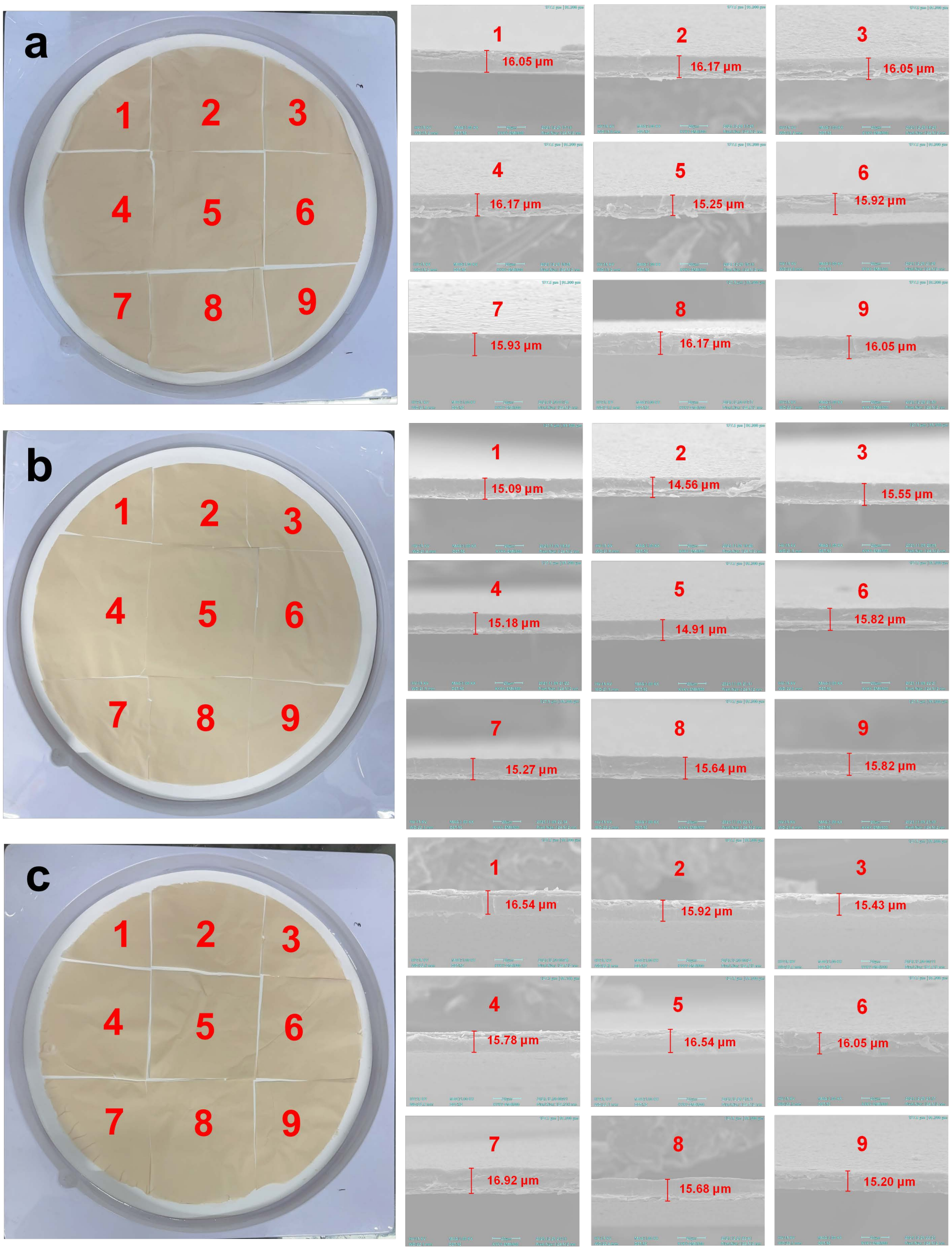

Supplementary Fig. 28 Photographs and cross-sectional SEM images of selected regions from large-area 2D-NNF films with the same preparation conditions. (a) 15.97±0.43 µm; (b) 15.32±0.48µm; (c)16.00±0.56 µm. Scale bar: 20 µm.

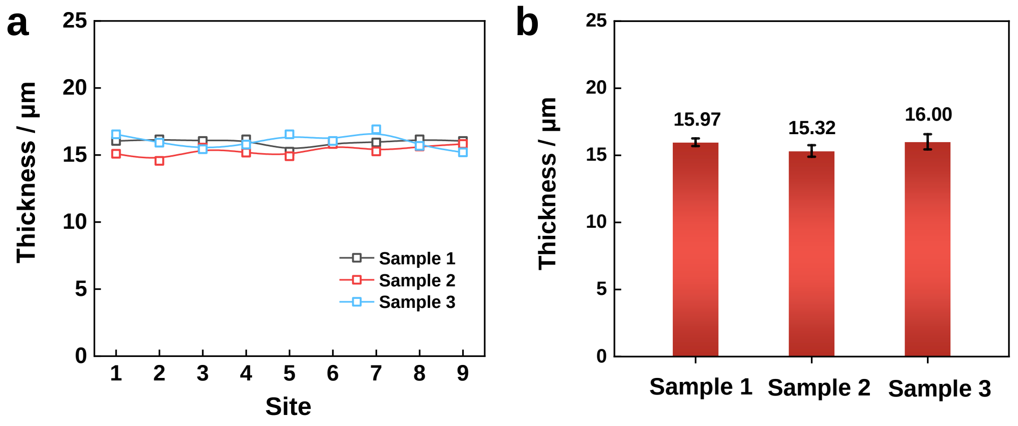

Supplementary Fig. 29 The thickness of different sites from different membranes with the same preparation conditions. (b) Average thickness of different membranes. The error bars in the figure represent the standard deviations for nine measurements.

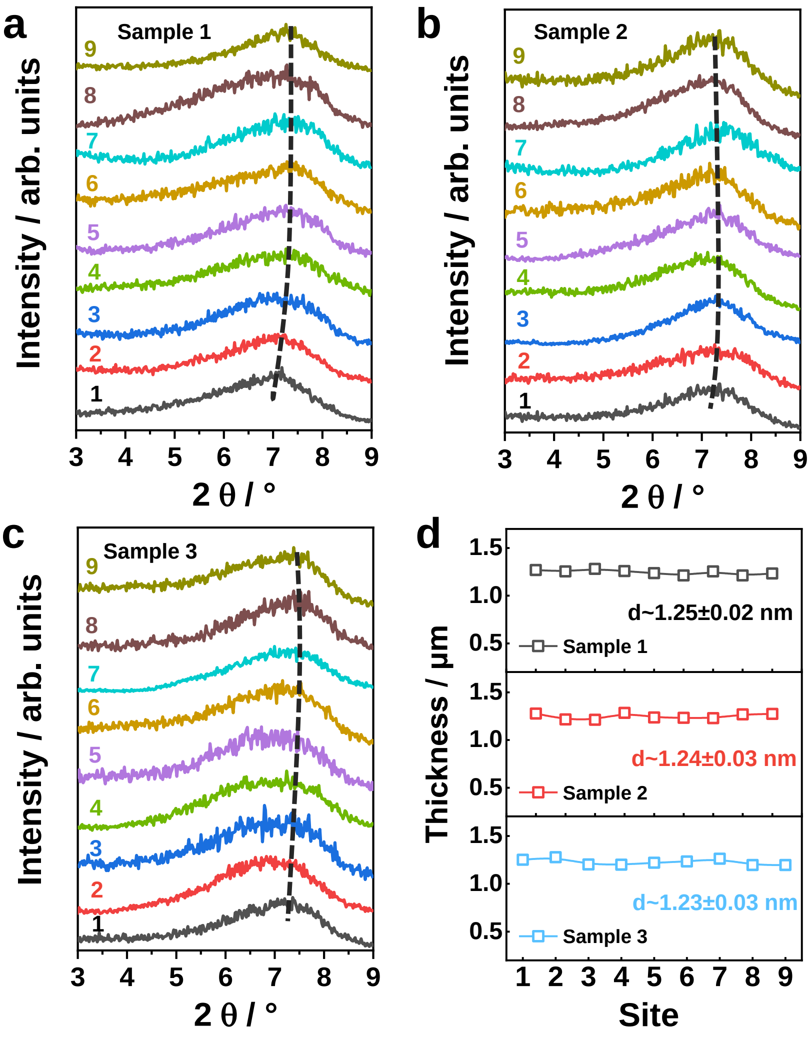

Supplementary Fig. 30 Small-angle XRD patterns of the different sites of large-area membranes with the same preparation conditions. Source data are provided as a Source Data file.

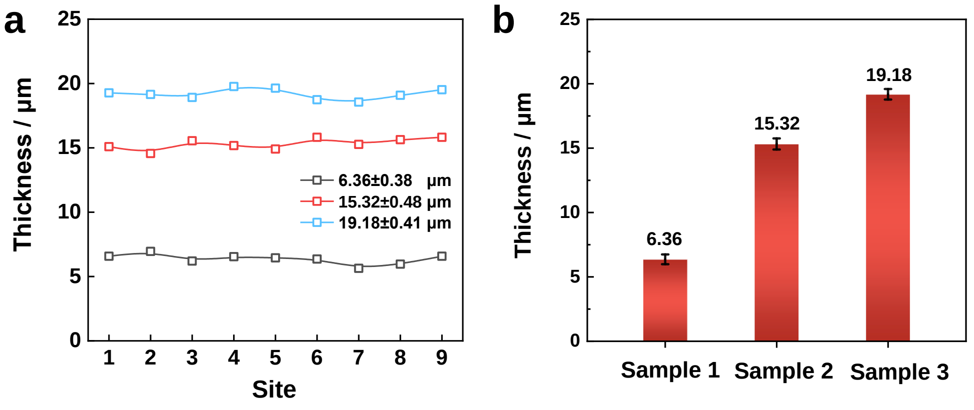

Supplementary Fig. 31 The thickness of different sites of the samples with different thickness. (b) Average thickness of different samples. The error bars in the figure represent the standard deviations for nine measurements.

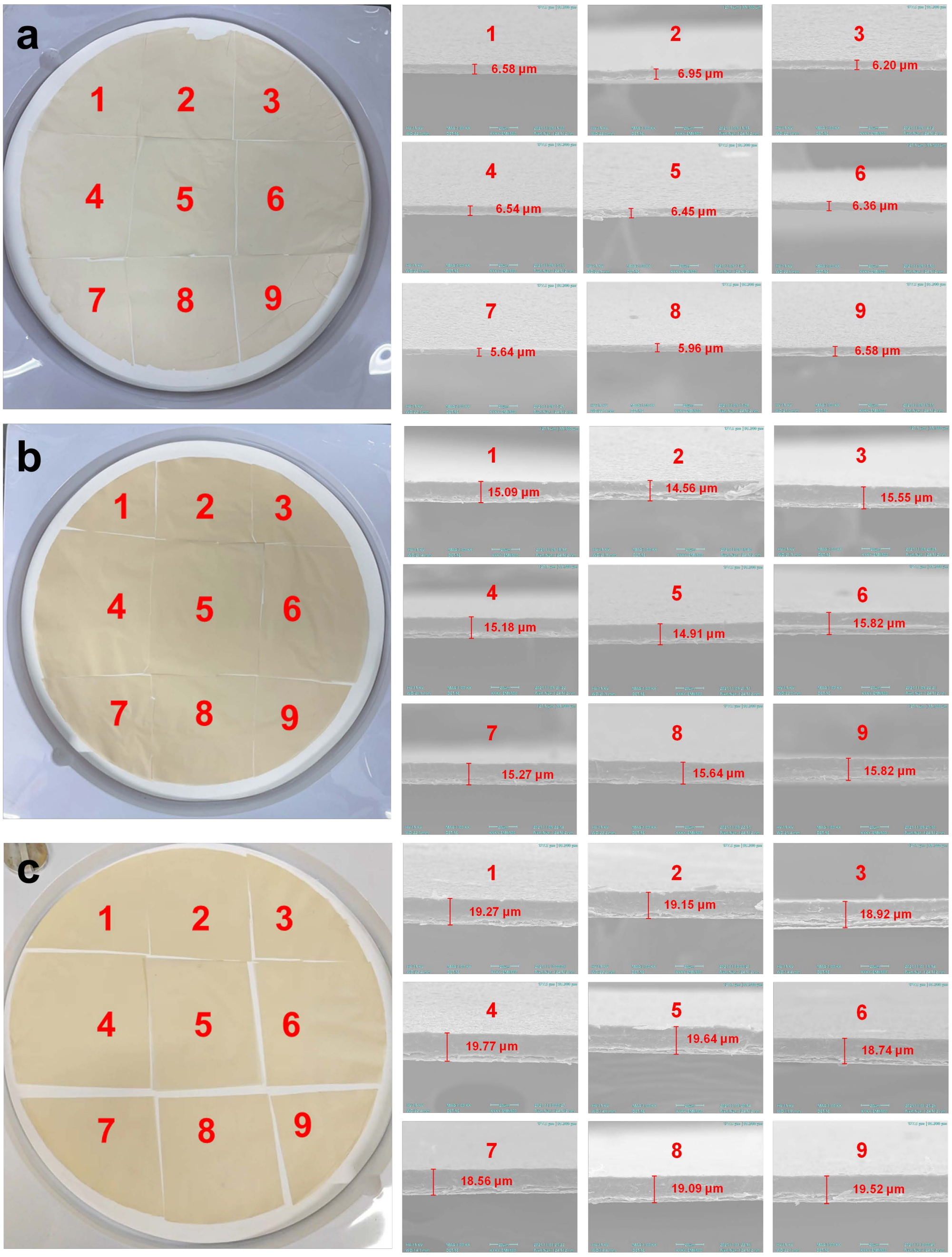

Supplementary Fig. 32 Photographs and cross-sectional SEM images of selected regions from large-area 2D-NNF films of different thicknesses (a) 6.36±0.38 μm; (b) 15.32±0.48μm; (c)19.18±0.41 μm. Scale bar: 20 μm.

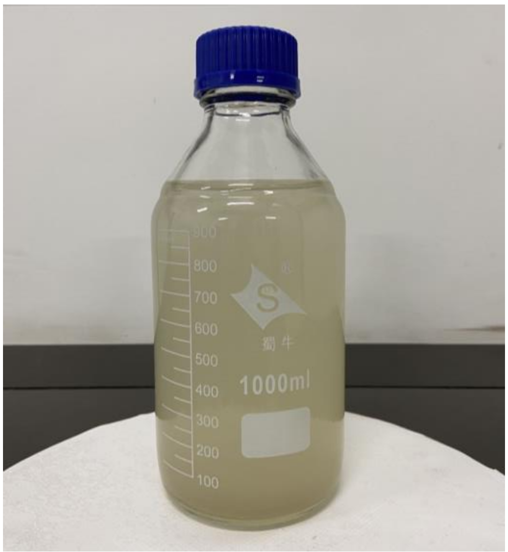

Supplementary Fig. 33 Photograph of the precursor solution of 2D-NNF.

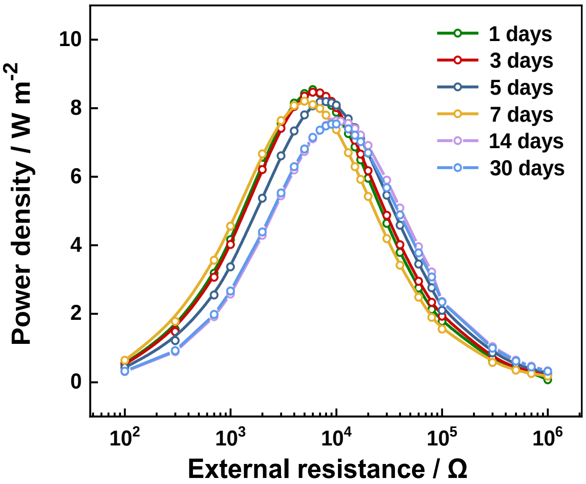

Supplementary Fig. 34 The power output stability of the 2D-NNF. Source data are provided as a Source Data file.

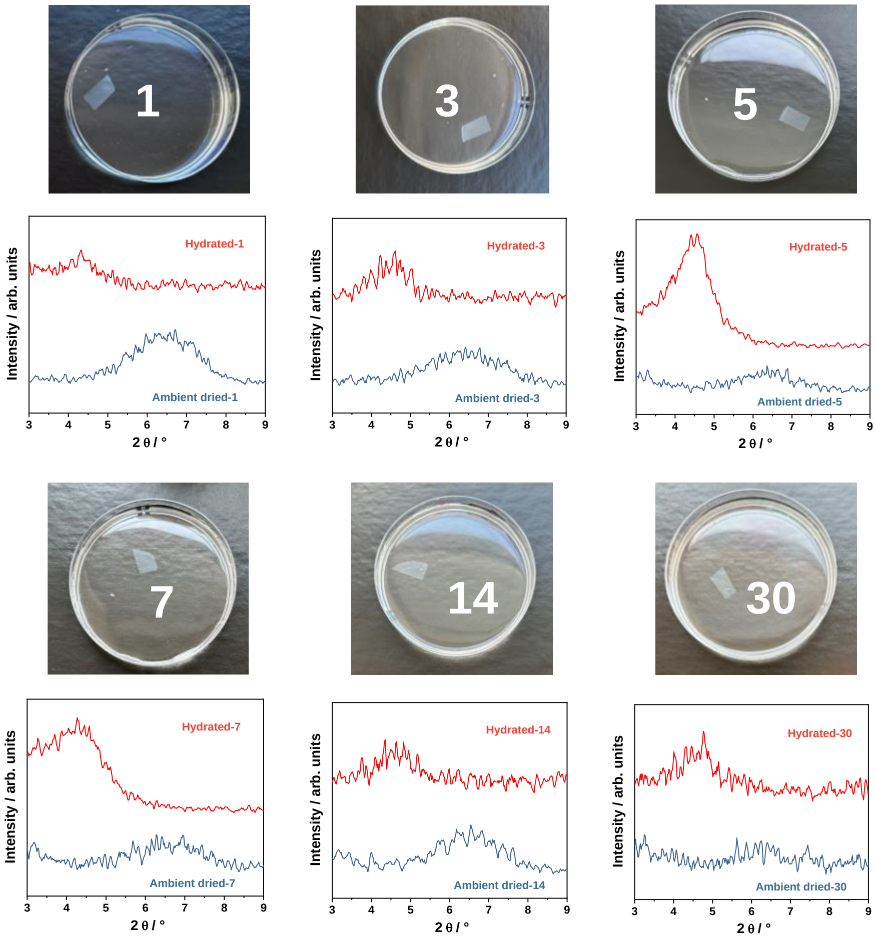

Supplementary Fig. 35 The stability of the 2D-NNF in aqueous solution and the XRD patterns of the nanofluidics after hydration and dried, indicating that the nanofluidics structure has a good reversible stability. Source data are provided as a Source Data file.

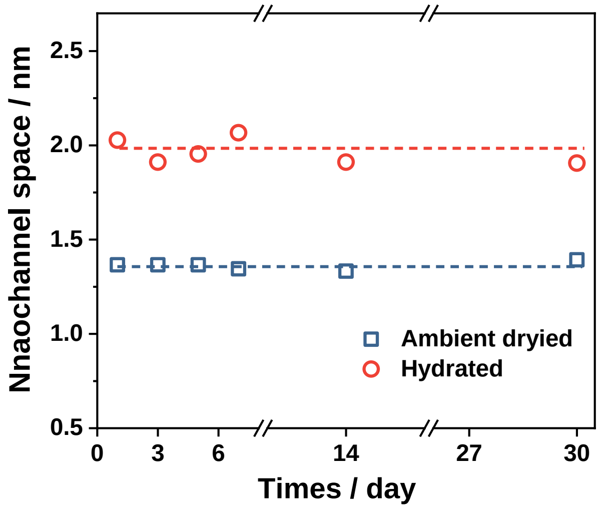

Supplementary Fig. 36 The change of nanochannel space of the 2D-NNF before and after hydration, implying that the 2D-NNF have a long-term stability in the osmotic energy harvesting. Source data are provided as a Source Data file.

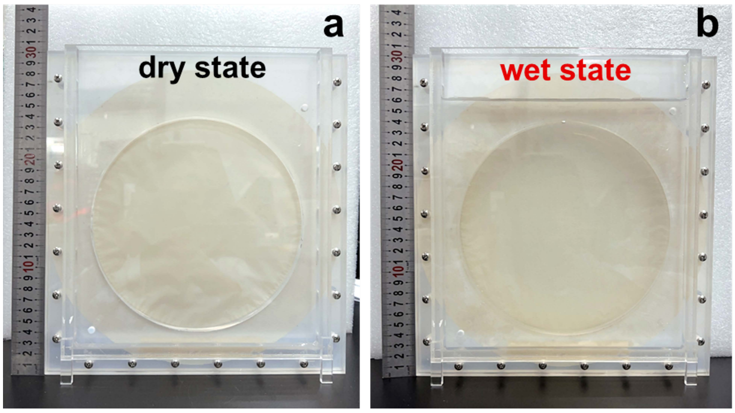

Supplementary Fig. 37 Special equipment for osmotic energy harvesting study of large-area 2D-NNF membrane (φ 30 cm). The self-standing membrane could be well immobilized in equipment for the subsequent measurements. After injecting solutions on both sides, the membrane could still maintain a good mechanical property, providing a good guarantee for long-term measurements.

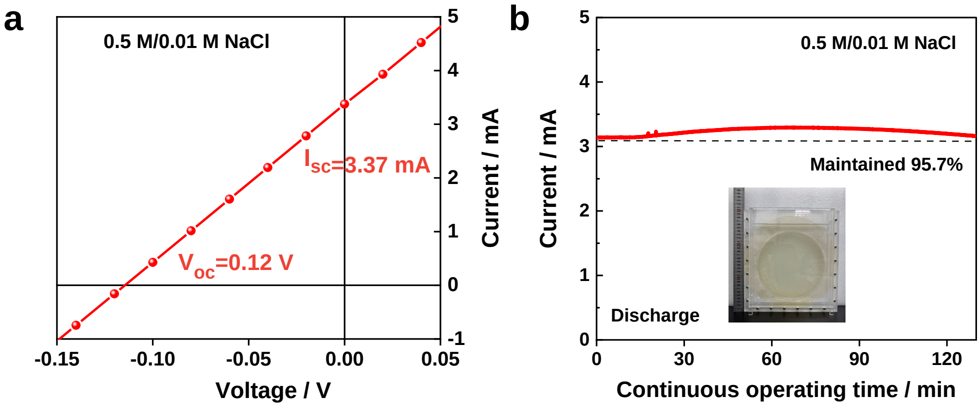

Supplementary Fig. 38 (a) Typical *I-V* curve of the large-area 2D-NNF with a diameter of 30 cm under the condition of simulated seawater/river salinity gradient (0.5 M/0.01 M NaCl). (b) Long-term current output of the large-area 2D-NNF membrane. Source data are provided as a Source Data file.

Supplementary Tables

Supplementary Table 1. Debye length varies with bulk solution concentration and pH values.

KCl concentration (mM) (pH~5.7)	Debye length λ_D (nm)	10 mM KCl (pH)	Debye length λ_D (nm)
0.1	30.5	3	2.9
1	9.6	5	3.1
10	3.1	7	3.1
100	1.0	9	3.1
1000	0.3	11	2.9

Supplementary Table 2. Average activity coefficients of various aqueous solutions with different ions and concentrations at 298 K.

Concentration/M	0.001	0.01	0.05	0.1	0.5	1
KCl	0.965	0.901	0.815	0.769	0.650	0.605
NaCl	0.966	0.904	0.823	0.778	0.682	0.658
LiCl	0.964	0.889	0.769	0.790	0.739	0.774
$CaCl_2$	0.887	0.724	0.574	0.518	0.448	0.500
$MgCl_2$	0.879	0.816	0.635	0.528	0.480	0.569

Supplementary Table 3. The corresponding values of V_{OS} and V_{thero} under different KCl electrolyte concentration gradient conditions.

Concentration gradient (mM/mM)	1/10	1/50	1/100	1/500	1/1000
V_{OS} (mV)	48	60	67	80	84
V_{thero} (mV)	57	96	112	150	165
Transference number (t^+)	0.92	0.81	0.80	0.77	0.75
Conversion efficiency η(%)	35	20	18	14	13

Supplementary Table 4. Ion migration rate (m s^{-1}) statistics in horizontal and vertical directions by a MD simulation.

Simulation time (ns)	11	12	13	14	15	Average rate
$V_{K}+$ (out)	3.29	3.22	3.17	3.3	3.27	3.25
$V_{K}+$ (in)	4.71	4.64	4.68	4.52	4.65	4.64
$V_{Cl}-$ (out)	0.33	0.28	0.38	0.37	0.44	0.36
$V_{Cl}-$ (in)	0.42	0.45	0.38	0.39	0.36	0.40
$H_{K}+$ (out)	1.47	1.55	1.42	1.58	1.68	1.54
$H_{K}+$ (in)	1.76	1.77	1.64	1.77	1.86	1.76
$H_{Cl}-$ (out)	1.64	1.58	1.62	1.66	1.7	1.64
$H_{Cl}-$ (in)	1.65	1.62	1.58	1.68	1.57	1.62

Here, “out” or “in” represents the positions of outer and inner pores of the nanosheet as shown in Supplementary Fig. 17, and the statistics of ion average migration rate are also shown in the picture.

Supplementary Table 5. Comparisons for key performance parameters of the 2D-NNF developed in this work with previousely reported single 1D, 2D and 3D nanofluidics. All the measurements were carried out by mixing artificial seawter and river water (0.5 M / 0.01 M NaCl).

Material system	Con-figuration	Power density (W/m^2)	Resistance (kΩ)	Thickness (μm)	Test area (mm^2)	Ref.
2D-NNF	2D	8.61	6	5.3	0.03	This work
Heterogeneous MXene	2D	8.6	10	4	0.03	21
iGOM	2D	6.78	30	10	0.03	22
GO/IL	2D	6.7	13	11	0.03	23
WS_2/ANF	2D	6.01	23	4	0.03	24
NBCM	2D	5.58	30	12	0.03	25
BN/ANF	2D	5.5	10	1	0.03	26
bsGOM	2D	5.5	36	4	0.03	27
Asymmetric GO	2D	5.32	30	5.87	0.04	28
MoS_2/CNF	2D	5.2	24	4	0.03	29
GO/SNF/GO	2D	5.07	40	5	0.03	30
GO/ANF	2D	5.06	20	2.2	0.03	16
Fusiform-GOMs	2D	4.94	26	2	0.03	31
MXene/CNF	2D	4.84	13	5	0.03	1
GO/CNF	2D	4.19	27	9	0.03	15
STFA-Na-MTM	2D	4.10	17	9.3	0.03	32
VMT	2D	4.1	15	2.1	0.03	33
GO/BP	2D	3.4	200	8	0.03	34
MXene/ANF	2D	3.7	27	4.5	0.03	14
MMT/ANF	2D	3.38	8	3	0.03	17
LDH/AAO	2D	2.85	23	45.8	0.03	35
MXene/BN	2D	2.3	60	10	0.03	36
Heterogeneous MXene	2D	2.3	15	3.5	0.03	37

Material system	Con-figuration	Power density (W/m^2)	Resistance (kΩ)	Thickness (μm)	Test area (mm^2)	Ref.
WS_2/CNF	2D	1.99	13	3	0.03	38
BP	2D	1.6	250	8	0.03	34
C-MXene/C-HNF	2D	1.09	17	—	0.03	39
Heterogeneous MUM	2D	0.7	30	16.5	0.2	40
GO/PPSU-Py	2D	0.76	300	31	0.03	41
MXene	2D	0.53	11	15	0.03	42
COF-$(SO_3Na)_1$/PAN	1D	97	8.2	0.053	0.00785	43
p-BCP-1	1D	19.3	10	—	0.008	44
TpEB@TpPa-SO_3Na	1D	19.2	5.5	0.5	0.03	45
ZnTPP-COF	1D	14.63	7	0.0011	0.006	46
TFPT-TMT COF	1D	13.3	17	0.3	0.00785	47
h-PEI	1D	13.2	15	0.012	0.008	5
PyPa-SO_3H COF/SANF	1D	8.7	5	7.2	0.03	48
PS-b-P2VP/MXene	1D	6.74	5.5	1.1	0.03	49
TpPa-SO_3H COF	1D	5.9	23	10.7	0.03	50
Cation-selective COF/AAO	1D	5.41	13	25	0.03	51
PAA-cPEI	1D	3.7	40	0.214	0.008	52
BDA-TAM	1D	2.96	17	1.5	0.02	53
Anion-selective COF/AAO	1D	2.5	5	38	0.03	54
BCP	1D	2.1	17	0.5	0.03	55
NMIM	3D	23	5	200	0.03	56
SPEEK/AAO/PPy	3D	9.65	2	2	0.03	57
MOF-on-MOF	3D	8.72	10	75.7	0.03	58
SPEEK-SPSF	3D	7	17	4.3	0.03	59
ZW-M7N1	3D	6.2	17	65	0.03	60
ICM-4	3D	6.18	20	—	0.03	61

Material system	Con-figuration	Power density (W/m^2)	Resistance (kΩ)	Thickness (μm)	Test area (mm^2)	Ref.
Anti-Swelling hydrogel	3D	6	10	50	0.03	[62]
HEMAP hydrogel	3D	5.38	15	25	0.03	[63]
PSS/HKUST-1/AAO	3D	5.2	—	0.2	0.03	[64]
MCS/AAO	3D	5.04	10	—	0.03	[65]
KANF	3D	4.8	10	4	0.03	[6]
ANF/Gel	3D	3.9	23	210	0.03	[66]
Carbon/AAO	3D	3.46	10	64	0.03	[67]
Hydrogel hybrid	3D	3.18	36	25	0.03	[68]
SNF/AAO	3D	2.86	23	65	0.03	[8]
CMWs	3D	2.78	13	135	0.03	[69]
PAEK-HS/PES-Py	3D	2.66	10	11	0.03	[70]
HENM	3D	2.22	10	—	0.03	[71]
TPPS/Al_2O_3	3D	2.16	10	25.2	0.03	[72]

Supplementary Table 6. Comparison with the two-dimensional (2D) materials nanofiber-based nanofluidics in the literatures (0.01 M/0.05 M NaCl).

Nanofluidic	Ion selectivity	Power density (W/m^2)	Resistance (kΩ)	Mechanical strength (MPa)	Conversion Efficiency (%)	Stability (days)	Ref
GO/CNF	0.82 (0.01-100 mM)	4.19	27	—	20	30	15
GO/ANF	0.77 (0.01-100 mM)	5.06	17	93	15	—	16
MXene/CNF	—	4.84	20	—	—	—	1
MXene/ANF	0.80 (0.1-500 mM)	3.7	27	101	18	30	14
MoS_2/CNF	0.90 (10-500 mM)	5.2	23	—	32	—	29
BN/ANF	0.80 (10-500 mM)	5.5	10	370	18	—	26
g-C_3N_4/CNF	—	0.15	—	65	—	30	73
WS_2/CNF	—	1.99		70	—	—	38
2D-NNF	0.82 (10-500 mM)	8.61	6	115	21	30	This work

Here, ion selectivity data are based on the maximum that can be reached at high concentrations (KCl or NaCl). The representative materials GO and MXene were selected for complete information comparison in the Fig. 5f.

Supplementary Table 7. Input and output inventory of each material preparation

Category	Input and Output	Unit	Process				
			MMT Preparation	GO Preparation	MXene Preparation	CNF Preparation	ANF Preparation
Energy	Electricity	MJ	1.08E+02	7.08E+02	1.11E+03	4.46E+02	9.77E+02
	Deionised water	kg	8.60E+03	1.04E+04	5.70E+03	5.90E+03	2.00E+01
	Bentonite clay	kg	3.00E+02				
	Sodium phosphate	kg	3.00E+00				
	Sodium hydroxide	kg		1.79E+01		2.02E+00	5.00E-01
	Sulfuric acid	kg		6.57E+02			2.50E+01
	Potassium permanganate	kg		5.22E+01			
	Hydrogen peroxide	kg		8.55E+02			
	Sodium nitrate	kg		7.15E+00			
	Hydrochloric acid	kg		1.17E+02	1.76E+02		
	Graphite	kg		1.79E+01	6.00E+00		
	Titanium	kg			9.00E+00		
Resource	Aluminum	kg			3.60E+00		
	Lithium fluoride	kg			1.49E+01		
	Hard wood	kg				1.20E+01	
	Sodium bromide	kg				6.00E–01	
	Sodium hypochlorite	kg				1.32E+00	
	Sodium sulfide	kg				8.60E–01	
	Calcium chloride	kg					2.40E+00
	Dimethylsulfid	kg					2.50E+02
	Benzal chloride	kg					2.00E+00
	N,N-dimenthylformamide	kg					4.00E+01
	Terephthaloyldichloride	kg					4.00E+00
	Potassium hydroxide	kg					5.00E+00
	MMT	kg	5.00E+00				
	GO	kg		5.00E+00			
Product	MXene	kg			5.00E+00		
	CNF	kg				5.00E+00	
	ANF	kg					5.00E+00
Pollutant	Waste water	t	3.70E+00	6.50E+00	7.00E–01	8.00E–02	1.40E–01

Supplementary Table 8. Comparison of environmental impact assessment characterization results of different two-dimensional materials and nanofiber materials.

Index	Units	MMT	GO	MXene	ANF	CNF
GWP	kg CO_2-Equiv.	7.60E+01	6.26E+02	4.72E+02	6.69E+02	6.66E+01
AP	kg SO_2-Equiv.	3.59E–01	4.68E+00	1.50E+00	1.36E+00	1.32E–01
ADP	kg MJ-Equiv.	1.03E+03	1.45E+04	6.45E+03	1.44E+04	1.15E+03
HTP	kg DCB-Equiv.	2.64E+00	4.08E+01	1.83E+02	2.13E+01	4.12E+00
MAETP	kg DCB-Equiv.	2.68E+03	3.17E+04	1.32E+05	1.65E+04	3.47E+03
FAETP	kg DCB-Equiv.	2.44E–01	3.35E+00	1.81E+00	1.88E+00	2.18E–01
POCP	kg Ethene-Equiv.	2.30E–02	2.66E–01	1.70E–01	1.13E–01	1.03E–02
EP	kg PO_4^{3-}-Equiv.	4.91E–02	1.40E–01	1.26E–01	1.50E–01	2.09E–02

Supplementary Table 9. The specific price of different materials involved in cost analysis.

Materials	Price ($/t)
Na^+-bentonite	188.4
natural graphite	652.2
Ti powder	34782.6
Al powder	14492.7
$(NaPO_3)_6$	1449.3
HCl	57.9
H_2SO_4	86.9
$NaNO_3$	507.2
$KMnO_4$	2681.2
H_2O_2	289.8
NaOH	521.7
NaBr	4057.9
NMP	2608.7
PPDA	4347.8
$CaCl_2$	231.9
DMSO	4347.8
TPC	2608.7
KOH	1594.2

Supplementary Table 10. Comparison with the mainstream 2D materials nanofiber-based nanofluidics from resource, environment and economy.

Nanofluidic Membrane	2D-NNF	GO/ANF	MXene/CNF	GO/CNF	MXene/ANF	MMT/ANF
GWP (kg CO_2e)	8.27E+01	7.60E+02	4.79E+02	6.70E+02	5.46E+02	1.53E+02
AP (kg SO_2e)	3.72E–01	4.81E+00	1.51E+00	4.77E+00	1.65E+00	5.22E–01
HTP (kg DCBe)	3.01E+00	4.51E+01	1.83E+02	4.35E+01	1.85E+02	5.20E+00
MAETP (kg DCBe)	3.03E+03	3.33E+04	1.35E+05	3.40E+04	1.34E+05	4.66E+03
FAETP (kg DCBe)	2.44E–01	3.35E+00	1.81E+00	3.49E+00	2.02E+00	4.70E–01
POCP (kg Ethenee)	2.40E–02	2.89E–01	1.71E–01	2.73E–01	1.82E–01	3.60E–02
EP (kg Phosphatee)	5.12E–02	1.70E–01	1.28E–01	1.54E–01	1.43E–01	6.71E–02
Energy consumption (kWh)	4.24E+01	2.51E+02	3.19E+02	2.79E+02	3.37E+02	6.26E+01
ADP fossil (MJ)	1.14E+03	1.59E+04	6.56E+03	1.53E+04	8.03E+03	2.76E+03
Corrosive reagent consumption (kg)	3.10E+00	9.94E+02	1.91E+02	9.40E+02	2.22E+02	3.74E+01
Cost (USD)	1.05E+02	3.84E+02	1.26E+03	3.83E+02	1.25E+03	1.30E+02

The above statistics are based on the final production of 5 kg of material (produce about 100 m^2 of membrane). Here, for the economic benefits, the cost in the production process of 2D-NNF is only 1/4 and 1/13 of GO and MXene nanofiber-based membrane, respectively, which can be attributed to the abundant reserves of natural bentonite, and primarily utilizes physical methods for substance purification and without the use of strong corrosive and oxidizing reagents. Consequently, for the environmental emissions, compared with GO fiber-based nanofluidics, 2D-NNF reduce the emissions of greenhouse gases (i.e., CO_2, CH_4) and acidic gases (i.e., SO_2) by about an order of magnitude during the production process. For resource consumption, the production process of 2D-NNF does not involve the consumption of non-renewable resource such as titanium and aluminium, as well as high-energy consumption such as ball milling and calcination. Therefore, its abiotic depletion potential (ADP) level and energy consumption are only 1/14 and 2/15 of MXene and GO nanofiber-based membrane, respectively.

Supplementary References

1 Liu, P. *et al.* Synergy of light and acid–base reaction in energy conversion based on cellulose nanofiber intercalated titanium carbide composite nanofluidics. *Energy Environ. Sci.* **14**, 4400-4409 (2021).

2 Sheng, N. *et al.* TEMPO-oxidized bacterial cellulose nanofibers/graphene oxide fibers for osmotic energy conversion. *ACS Appl. Mater. Interfaces.* **13**, 22416-22425 (2021).

3 Li, T. *et al.* A nanofluidic ion regulation membrane with aligned cellulose nanofibers. *Sci. Adv.* **5**, eaau4238 (2019).

4 Daiguji, H. Ion transport in nanofluidic channels. *Chem. Soc. Rev.* **39**, 901-911 (2010).

5 Li, C. *et al.* Large-scale, robust mushroom-shaped nanochannel array membrane for ultrahigh osmotic energy conversion. *Sci. Adv.* **7**, eabg2183 (2021).

6 Ding, L. *et al.* Ultrathin and ultrastrong kevlar aramid nanofiber membranes for highly stable osmotic energy conversion. *Adv. Sci.* **9**, 2202869 (2022).

7 Mei, Y. & Tang, C. Y. Recent developments and future perspectives of reverse electrodialysis technology: A review. *Desalination* **425**, 156-174 (2018).

8 Xin, W. *et al.* High-performance silk-based hybrid membranes employed for osmotic energy conversion. *Nat. Commun.* **10**, 1-10 (2019).

9 Ge, P., Wang, H.-j., Zhao, J., Xie, L. & Zhang, Q. Preparation of high purity graphite by an alkaline roasting-leaching method. *Carbon* **7**, 2123-2124 (2010).

10 Zhang, T. *et al.* Synthesis of two-dimensional $Ti_3C_2T_x$ MXene using HCl+LiF etchant: enhanced exfoliation and delamination. *J. Alloys Compd* **695**, 818-826 (2017).

11 Wang, Z. et al. The purification process of bentonite. *Modern Minin* **03**, 26-29 (2013).

12 Saito, T., Kimura, S., Nishiyama, Y. & Isogai, A. Cellulose nanofibers prepared by TEMPO-mediated oxidation of native cellulose. *Biomacromolecules* **8**, 2485-2491 (2007).

13 Wu, G. c., Tanaka, H., Sanui, K. & Ogata, N. Synthesis of aromatic polyamide by a direct polycondensation with triphenylphosphine. *J Polym Sci: Polymer Letters Edition* **19**, 343-346 (1981).

14 Zhang, Z. *et al.* Mechanically strong MXene/Kevlar nanofiber composite membranes as high-performance nanofluidic osmotic power generators. *Nat. Commun.* **10**, 1-9 (2019).

15 Wu, Y. *et al.* Enhanced ion transport by graphene oxide/cellulose nanofibers assembled membranes for high-performance osmotic energy harvesting. *Mater. Horiz.* **7**, 2702-2709 (2020).

16 Chen, J. *et al.* Biomimetic nanocomposite membranes with ultrahigh ion selectivity for osmotic power conversion. *ACS Cent. Sci.* **7**, 1486-1492 (2021).

17 Qin, R. *et al.* Nanofiber-reinforced clay-based 2D nanofluidics for highly efficient osmotic energy harvesting. *Nano Energy* **100**, 107526 (2022).

18 Tombácz, E. & Szekeres, M. Colloidal behavior of aqueous montmorillonite suspensions: the specific role of pH in the presence of indifferent electrolytes. *Appl Clay Sci* **27**, 75-94, (2004).

19 Kaufhold, S. & Dohrmann, R. The variable charge of dioctahedral smectites. *J. Colloid Interface Sci.* **390**, 225-233, (2013).

20 Pecini, E. M. & Avena, M. J. Measuring the isoelectric point of the edges of clay mineral particles: The case of montmorillonite. *Langmuir* **29**, 14926-14934, (2013).

21 Ding, L. *et al.* Bioinspired $Ti_3C_2T_x$ MXene-based ionic diode membrane for high-efficient osmotic energy conversion. *Angew. Chem. Int. Ed.* **61**, e202206152 (2022).

22 Yan, P. P. *et al.* Two-dimensional nanofluidic membranes with intercalated in-plane shortcuts for high-performance blue energy harvesting. *Small* **19**, 2205003 (2023).

23 Hu, Y. *et al.* Confined ionic-liquid-mediated cation diffusion through layered membranes for high-performance osmotic energy conversion. *Adv. Mater.,* 2301285 (2023).

24 Wang, Q. *et al.* Efficient solar-osmotic power generation from bioinspired anti-fouling 2D WS2 composite membranes. *Angew. Chem. Int. Ed.* **62**, e202302938 (2023).

25 Zhang, M. *et al.* Enhanced selective ion transport by assembling nanofibers to membrane pairs with channel-like nanopores for osmotic energy harvesting. *Nano Energy* **103**, 107786 (2022).

26 Chen, C. *et al.* Bio-inspired nanocomposite membranes for osmotic energy harvesting. *Joule* **4**, 247-261 (2020).

27 Qian, Y. *et al.* Boosting osmotic energy conversion of graphene oxide membranes via self-exfoliation behavior in nano-confinement spaces. *J. Am. Chem. Soc.* **144**, 13764-13772 (2022).

28 Bang, K. R., Kwon, C., Lee, H., Kim, S. & Cho, E. S. Horizontally asymmetric nanochannels of graphene oxide membranes for efficient osmotic energy harvesting. *ACS Nano* **17**, 10000-10009 (2023).

29 Zhu, C. *et al.* Metallic two-dimensional MoS_2 composites as high-performance osmotic energy conversion membranes. *J. Am. Chem. Soc.* **143**, 1932-1940 (2021).

30 Xin, W. *et al.* Biomimetic nacre-like silk-crosslinked membranes for osmotic energy harvesting. *ACS Nano* **14**, 9701-9710 (2020).

31 Qian, Y. *et al.* Two-dimensional membranes with highly charged nanochannels for osmotic energy conversion. *ChemSusChem* **15**, e202200933 (2022).

32 Ding, Z. *et al.* Promoting osmotic energy conversion through fluorinated nanochannel membranes with large-scale exfoliation and low transmission resistance. *J. Mater. Chem. A* **11**, 8798-8808 (2023).

33 Cao, L. *et al.* Lamellar porous vermiculite membranes for boosting nanofluidic osmotic energy conversion. *J. Mater. Chem. A.* **9**, 14576-14581 (2021).

34 Zhang, Z. *et al.* Oxidation promoted osmotic energy conversion in black phosphorus membranes. *Proc. Natl. Acad. Sci. U.S.A.* **117**, 13959-13966 (2020).

35 Liu, Y., Ping, J. & Ying, Y. Anion-selective layered double hydroxide composites-based osmotic energy conversion for real-time nutrient solution detection. *Adv. Sci.* **9**, 2103696

(2022).

36 Yang, G. *et al.* Stable $Ti_3C_2T_x$ MXene–boron nitride membranes with low internal resistance for enhanced salinity gradient energy harvesting. *ACS Nano* **15**, 6594-6603 (2021).

37 Wang, J. *et al.* Heterogeneous two-dimensional lamellar $Ti_3C_2T_x$ membrane for osmotic power harvesting. *Chem. Eng. J.* **452**, 139531 (2023).

38 Gao, Z. *et al.* Design of metallic phase WS_2/cellulose nanofibers composite membranes for light-boosted osmotic energy conversion. *Carbohydr. Polym.* **296**, 119847 (2022).

39 Rao, J. *et al.* Nacre-inspired mechanically robust films for osmotic energy conversion. *Adv. Funct. Mater.,* 2309869 (2023).

40 Wei, C. *et al.* Parallel arrays of clay nanosheets sandwiched in two-dimensional nanofluidic membrane for enhanced ion transport properties. *J. Membr. Sci.* **680**, 121744 (2023).

41 Zhu, X. *et al.* A charge-density-tunable three/two-dimensional polymer/graphene oxide heterogeneous nanoporous membrane for ion transport. *ACS Nano* **11**, 10816-10824 (2017).

42 Liu, P. *et al.* Neutralization reaction assisted chemical-potential-driven ion transport through layered titanium carbides membrane for energy harvesting. *Nano Lett.* **20**, 3593-3601 (2020).

43 Zuo, X. *et al.* Thermo-osmotic energy conversion enabled by covalent-organic-framework membranes with record output power density. *Angew. Chem. Int. Ed.* **61**, e202116910 (2022).

44 Li, C. *et al.* One porphyrin per chain self-assembled helical ion-exchange channels for ultrahigh osmotic energy conversion. *J. Am. Chem. Soc.* **144**, 9472-9478 (2022).

45 Cao, L. *et al.* An ionic diode covalent organic framework membrane for efficient osmotic energy conversion. *ACS Nano* **16**, 18910-18920 (2022).

46 Yang, J. *et al.* Advancing osmotic power generation by covalent organic framework monolayer. *Nat. Nanotechnol.* **17**, 622-628 (2022).

47 Wang, K. *et al.* Monolayer-assisted surface-initiated schiff-base-mediated aldol polycondensation for the synthesis of crystalline sp2 carbon-conjugated covalent organic framework thin films. *J. Am. Chem. Soc.* **145**, 5203-5210 (2023).

48 Man, Z. *et al.* Serosa-mimetic nanoarchitecture membranes for highly efficient osmotic energy generation. *J. Am. Chem. Soc.* **143**, 16206-16216 (2021).

49 Lin, X. *et al.* Heterogeneous MXene/PS-b-P2VP nanofluidic membranes with controllable ion transport for osmotic energy conversion. *Adv. Funct. Mater.* **31**, 2105013 (2021).

50 Hou, S. *et al.* Free-standing covalent organic framework membrane for high-efficiency salinity gradient energy conversion. *Angew. Chem. Int. Ed.* **133**, 10013-10018 (2021).

51 Gao, M. *et al.* A bioinspired ionic diode membrane based on sub-2 nm covalent organic framework channels for ultrahigh osmotic energy generation. *Nano Energy* **105**, 108007 (2023).

52 Yang, X. *et al.* Enhanced osmotic energy conversion through an asymmetric nanochannel array membrane with an ultrathin selective layer. *Chem. Mater.* **35**, 7266-7272 (2023).
53 Wang, C. *et al.* Ultrathin self-standing covalent organic frameworks toward highly-efficient nanofluidic osmotic energy generator. *Adv. Funct. Mater.* **32**, 2204068 (2022).
54 Chen, M. *et al.* In situ growth of imine-bridged anion-selective COF/AAO membrane for ion current rectification and nanofluidic osmotic energy conversion. *Adv. Funct. Mater.*, 2302427 (2023).
55 Zhang, Z. *et al.* Ultrathin and ion-selective janus membranes for high-performance osmotic energy conversion. *J. Am. Chem. Soc.* **139**, 8905-8914 (2017).
56 Zhang, F., Yu, J., Si, Y. & Ding, B. Meta-aerogel ion motor for nanofluid osmotic energy harvesting. *Adv. Mater.,* 2302511 (2023).
57 Hao, J. *et al.* A euryhaline-fish-inspired salinity self-adaptive nanofluidic diode leads to high-performance blue energy harvesters. *Adv. Mater.* **34**, 2203109 (2022).
58 Tonnah, R. K. *et al.* Bioinspired angstrom-scale heterogeneous MOF-on-MOF membrane for osmotic energy harvesting. *ACS Nano* **17**, 12445-12457 (2023).
59 Zhao, X. *et al.* Metal organic framework enhanced SPEEK/SPSF heterogeneous membrane for ion transport and energy conversion. *Nano Energy* **81**, 105657 (2021).
60 Sun, Y. *et al.* Tailoring a poly (ether Sulfone) bipolar membrane: osmotic-energy generator with high power density. *Angew. Chem. Int. Ed.* **132**, 17576-17581 (2020).
61 Chen, W. *et al.* Ionic crosslinking-induced nanochannels: nanophase separation for ion transport promotion. *Adv. Mater.* **34**, 2108410 (2022).
62 Bian, G. *et al.* Anti-swelling gradient polyelectrolyte hydrogel membranes as high-performance osmotic energy generators. *Angew. Chem. Int. Ed.* **133**, 20456-20462 (2021).
63 Chen, W. *et al.* Improved ion transport and high energy conversion through hydrogel membrane with 3D interconnected nanopores. *Nano Lett.* **20**, 5705-5713 (2020).
64 Pan, S. *et al.* Toward scalable nanofluidic osmotic power generation from hypersaline water sources with a metal–organic framework membrane. *Angew. Chem. Int. Ed.* **62**, e202218129 (2023).
65 Zhou, S. *et al.* Interfacial super-assembly of ordered mesoporous carbon-silica/AAO hybrid membrane with enhanced permselectivity for temperature-and pH-Sensitive smart ion transport. *Angew. Chem. Int. Ed.* **133**, 26371-26380 (2021).
66 Zhang, Z. *et al.* Improved osmotic energy conversion in heterogeneous membrane boosted by three-dimensional hydrogel interface. *Nat. Commun.* **11**, 1-8 (2020).
67 Gao, J. *et al.* High-performance ionic diode membrane for salinity gradient power generation. *J. Am. Chem. Soc.* **136**, 12265-12272 (2014).
68 Chen, W. *et al.* Improved ion transport in hydrogel-based nanofluidics for osmotic energy conversion. *ACS Cent. Sci.* **6**, 2097-2104 (2020).
69 Xie, L. *et al.* Sequential superassembly of nanofiber arrays to carbonaceous ordered

mesoporous nanowires and their heterostructure membranes for osmotic energy conversion. *J. Am. Chem. Soc.* **143**, 6922-6932 (2021).

70 Zhu, X. *et al.* Unique ion rectification in hypersaline environment: A high-performance and sustainable power generator system. *Sci. Adv.* **4**, eaau1665 (2018).

71 Ling, H. *et al.* Heterogeneous electrospinning nanofiber membranes with pH-regulated ion gating for tunable osmotic power harvesting. *Angew. Chem. Int. Ed.* **135**, e202212120 (2023).

72 Zhang, D., Ren, Y., Fan, X., Zhai, J. & Jiang, L. Photoassisted salt-concentration-biased electricity generation using cation-selective porphyrin-based nanochannels membrane. *Nano Energy* **76**, 105086 (2020).

73 Gao, Z. *et al.* Increased ion transport and high-efficient osmotic energy conversion through aqueous stable graphitic carbon nitride/cellulose nanofiber composite membrane. *Carbohydr. Polym.* **280**, 119023 (2022).
