# Supplementary material for: All-natural 2D nanofluidics as highly-efficient osmotic energy generators
Source: Nat Commun. 2024 Apr 29;15:3649. doi: 10.1038/s41467-024-47915-z (PMC11058229; doi:10.1038/s41467-024-47915-z)
Supplement: Supplementary file 3 — Reporting Summary [file 41467_2024_47915_MOESM3_ESM.pdf]

nature portfolio

Corresponding author(s): zhangqianqian@bjut.edu.cn

Last updated by author(s): Feb 12, 2024

Reporting Summary

Nature Portfolio wishes to improve the reproducibility of the work that we publish. This form provides structure for consistency and transparency in reporting. For further information on Nature Portfolio policies, see our Editorial Policies and the Editorial Policy Checklist.

Statistics

For all statistical analyses, confirm that the following items are present in the figure legend, table legend, main text, or Methods section.

n/a	Confirmed	
☐	☒	The exact sample size (*n*) for each experimental group/condition, given as a discrete number and unit of measurement
☐	☒	A statement on whether measurements were taken from distinct samples or whether the same sample was measured repeatedly
☒	☐	The statistical test(s) used AND whether they are one- or two-sided<br>*Only common tests should be described solely by name; describe more complex techniques in the Methods section.*
☐	☒	A description of all covariates tested
☐	☒	A description of any assumptions or corrections, such as tests of normality and adjustment for multiple comparisons
☐	☒	A full description of the statistical parameters including central tendency (e.g. means) or other basic estimates (e.g. regression coefficient) AND variation (e.g. standard deviation) or associated estimates of uncertainty (e.g. confidence intervals)
☒	☐	For null hypothesis testing, the test statistic (e.g. *F*, *t*, *r*) with confidence intervals, effect sizes, degrees of freedom and *P* value noted<br>*Give P values as exact values whenever suitable.*
☒	☐	For Bayesian analysis, information on the choice of priors and Markov chain Monte Carlo settings
☐	☒	For hierarchical and complex designs, identification of the appropriate level for tests and full reporting of outcomes
☒	☐	Estimates of effect sizes (e.g. Cohen's *d*, Pearson's *r*), indicating how they were calculated

Our web collection on statistics for biologists contains articles on many of the points above.

Software and code

Policy information about availability of computer code

Data collection	No specialized software was used in data collection.
Data analysis	The molecular dynamics (MD) simulation in this study uses GROMACS (version 2020.3_GPU) software Berendsen thermostat, and the coupling time constant is 0.5 ps. The periodic boundary conditions and the ClayFF force field are used for calculation, the long-range electrostatic Particle-Mesh Ewald (PME) electrostatic calculation method is adopted. In this study, the short-range electrostatic and van der Waals cut off distance are set to 1 nm, and the concentration of K+ and Cl ions is set to 1 mol L1. The transport of ions and charges is theoretically studied by MD simulation. The first is to establish a system with the theme of aqueous solution, at which time no ions exist. Step 2, replace some water molecules according to the concentration required by the experiment to make the ion concentration in the solution consistent with the experimental value, and set it as 1 mol L1. After NVT (N: number of particles, v: volume, t: temperature) balance at 450 K, NPT (N: number of particles, p: pressure, t: temperature) balance at 300 K for 5 ns annealing. The last step is to statistically analyze the ion migration number, ion migration speed and binding with VMD.

For manuscripts utilizing custom algorithms or software that are central to the research but not yet described in published literature, software must be made available to editors and reviewers. We strongly encourage code deposition in a community repository (e.g. GitHub). See the Nature Portfolio guidelines for submitting code & software for further information.

Data

Policy information about availability of data

All manuscripts must include a data availability statement. This statement should provide the following information, where applicable:

- Accession codes, unique identifiers, or web links for publicly available datasets
- A description of any restrictions on data availability
- For clinical datasets or third party data, please ensure that the statement adheres to our policy

All data supporting the research in this study are available within the article and Supplementary Information file. Source data are provided with this paper. The source data can also be accessed through the Figshare repository and are freely available for download.

Research involving human participants, their data, or biological material

Policy information about studies with human participants or human data. See also policy information about sex, gender (identity/presentation), and sexual orientation and race, ethnicity and racism.

Reporting on sex and gender	N/A
Reporting on race, ethnicity, or other socially relevant groupings	N/A
Population characteristics	N/A
Recruitment	N/A
Ethics oversight	N/A

Note that full information on the approval of the study protocol must also be provided in the manuscript.

Field-specific reporting

Please select the one below that is the best fit for your research. If you are not sure, read the appropriate sections before making your selection.

☐ Life sciences ☐ Behavioural & social sciences ☒ Ecological, evolutionary & environmental sciences

For a reference copy of the document with all sections, see nature.com/documents/nr-reporting-summary-flat.pdf

Ecological, evolutionary & environmental sciences study design

All studies must disclose on these points even when the disclosure is negative.

Study description	A two-dimensional all-natural nanofluidic (2D-NNF) is developed as a robust and highly-efficient osmotic energy generator based on an interlocking configuration of stacked montmorillonite nanosheets (from natural clay) and their intercalated cellulose nanofibers (from natural wood).
Research sample	All-natural 2D nanofluidics membrane and the as-assembled osmotic energy conversion system based on it.
Sampling strategy	The osmotic energy conversion was carried out by selecting several test sites from the different regions of membrane under the same test conditions. The thickness of the membrane was calculated from the SEM images using a microscopic image analysis. Each kind of the sample was tested multiple times in different areas to ensure the accuracy of sample thickness.
Data collection	Data collection was performed using the standard method in this type of work. Details are provided in the Methods section of the manuscript.
Timing and spatial scale	Data collection was performed at predetermined times.
Data exclusions	No data were excluded from the analyses.
Reproducibility	Each experimental characterization was repeated independently for at least three times with similar results.
Randomization	The MMT-based nanofluidic membrane with different CNF content were randomly divided into four groups: 0 wt%, 5wt%, 10 wt%, 15wt% and 20 wt%. The layer spacing and osmotic energy conversion performance of the samples were investigated respectively. When analyzing the osmotic energy power generation potential of large-area membranes, multiple groups of samples were constructed on the basis of a fixed CNF content of 10 wt%. Each group of samples was randomly selected for test points, and the structure and morphology characterization tests and osmotic energy conversion tests were conducted under the same test conditions to ensure the objectivity of results and reduce the possibility of selection bias.

Blinding	Blinding was not applicable to this study as there was no experiments involving specific treatments or groups.

Did the study involve field work? ☐ Yes ☒ No

Reporting for specific materials, systems and methods

We require information from authors about some types of materials, experimental systems and methods used in many studies. Here, indicate whether each material, system or method listed is relevant to your study. If you are not sure if a list item applies to your research, read the appropriate section before selecting a response.

Materials & experimental systems

n/a	Involved in the study
☒	☐ Antibodies
☒	☐ Eukaryotic cell lines
☒	☐ Palaeontology and archaeology
☒	☐ Animals and other organisms
☒	☐ Clinical data
☒	☐ Dual use research of concern
☒	☐ Plants

Methods

n/a	Involved in the study
☒	☐ ChIP-seq
☒	☐ Flow cytometry
☒	☐ MRI-based neuroimaging

Plants

Seed stocks	N/A
Novel plant genotypes	N/A
Authentication	N/A
